# Plant functional types broadly describe water use strategies in the Caatinga, a seasonally dry tropical forest in northeast Brazil

**DOI:** 10.1002/ece3.7949

**Published:** 2021-08-04

**Authors:** Cynthia L. Wright, André L. A. de Lima, Eduardo S. de Souza, Jason B. West, Bradford P. Wilcox

**Affiliations:** ^1^ Environmental Sciences Division Oak Ridge National Laboratory Oak Ridge TN USA; ^2^ Ecology and Conservation Biology Texas A&M University College Station TX USA; ^3^ Universidade Federal Rural de Pernambuco/Unidade Acadêmica de Serra Talhada (UFRPE/UAST) Serra Talhada Brasil

**Keywords:** drought stress and tolerance, ecophysiology, leaf water potential, phenology and wood density, plant functional types, seasonally dry tropical forests, stable isotope ratios, water use strategies and resource acquisition trade‐offs

## Abstract

1. In seasonally dry tropical forests, plant functional type can be classified as deciduous low wood density, deciduous high wood density, or evergreen high wood density species. While deciduousness is often associated with drought‐avoidance and low wood density is often associated with tissue water storage, the degree to which these functional types may correspond to diverging and unique water use strategies has not been extensively tested.
2. We examined (a) tolerance to water stress, measured by predawn and mid‐day leaf water potential; (b) water use efficiency, measured via foliar δ^13^C; and (c) access to soil water, measured via stem water δ^18^O.
3. We found that deciduous low wood density species maintain high leaf water potential and low water use efficiency. Deciduous high wood density species have lower leaf water potential and variable water use efficiency. Both groups rely on shallow soil water. Evergreen high wood density species have low leaf water potential, higher water use efficiency, and access alternative water sources. These findings indicate that deciduous low wood density species are drought avoiders, with a specialized strategy for storing root and stem water. Deciduous high wood density species are moderately drought tolerant, and evergreen high wood density species are the most drought tolerant group.
4. *Synthesis*. Our results broadly support the plant functional type framework as a way to understand water use strategies, but also highlight species‐level differences.

## Graphical abstract

Plant functional types in the Caatinga seasonally dry forests can be defined according to phenology and wood density. Our work demonstrates that there is a tight correspondence between these plant function types and water use strategies.

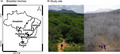

## 1 INTRODUCTION

Classification by plant functional type (PFT) simplifies biodiversity by grouping species according to their response to specific environmental conditions or disturbances (Lavorel et al., 1997). The emergence of functional trait spectra, such as the leaf, wood, and root economic spectra (Chave et al., 2006; Reich, 2014; Wright et al., 2004) means that PFTs should represent unique functions, and that the coordination of traits could reflect the trade‐offs associated with acquiring and allocating limited resources (Díaz et al., 2016; Méndez‐Alonzo et al., 2012). Seasonally dry tropical forests (SDTFs) are defined by four to seven months of severe water shortage (Bullock et al., 1995; Dirzo et al., 2011). When periods of water abundance alternate with periods of scarcity, species must balance trade‐offs between carbon gain and water conservation. Ecological strategies which are more acquisitive maximize carbon gain at the cost of water loss, and strategies which are more conservative optimize water use at the cost of carbon gain (Pineda‐García et al., 2013; Schwinning & Ehleringer, 2010). Seasonal conditions combined with a relatively high biodiversity make SDTFs an ideal biome in which to explore a range of species responses that enable successful establishment and coexistence (Esquivel‐Muelbert et al., 2017; Lebrija‐Trejos et al., 2010; Lohbeck et al., 2013). Additionally, understanding the coherence of PFT to plant physiological function and ecological strategy has become increasingly important for utilizing and refining global vegetation models, although species‐specific differences are often largely ignored (Fisher et al., 2018; Scheiter et al., 2013).

Plant functional types can be defined by multiple combinations of traits that reflect plant physiological function and ecological strategy. In SDTFs specifically, PFTs are often defined according to wood density and phenology (Borchert, 1994; de Lima et al., 2012; Oliveira et al., 2015) because of the link to water use. In the dry tropics, wood density has been negatively correlated with stem hydraulic conductance (Markesteijn et al., 2011), stomatal conductance, and daily transpiration (Bucci et al., 2004; Worbes et al., 2013). High wood density often means a thicker xylem vessel wall and higher vessel density, and therefore more negative leaf water potentials and greater resistance to cavitation and embolism (Hacke et al., 2001; Pineda‐García et al., 2015; Gelder et al., 2006). Such trees may have loose stomatal control, or anisohydric regulation of water status, and narrower hydraulic safety margins (McDowell et al., 2008). High wood density can also mean smaller vessel area and reduced water storage and capacitance (Carrasco et al., 2014; Chave et al., 2006). Thus, we expect high wood density species to be tolerant of drought conditions and to be associated with conservative strategies.

Many of the low wood density species in SDTFs are stem succulents (Ávila‐Lovera & Ezcurra, 2016). These have significantly larger vessel areas, lower vessel density, greater water storage capacity (Pineda‐García et al., 2015; Pratt & Jacobsen, 2017), and higher water transport capability (Chave et al., 2006; Zanne et al., 2010) than nonsucculent trees. Low wood density is also associated with a low resistance to cavitation, as related to poor mechanical strength or larger vessel area (Jacobsen et al., 2005; Lachenbruch & McCulloh, 2014; Venturas et al., 2017). Stem succulents also have a low tolerance to negative leaf water potentials, experience mid‐day drops in stomatal conductance, and are subject to early onset of leaf fall (Butz et al., 2017; Worbes et al., 2013)—although their high capacity for water storage may help buffer changes in xylem pressure and leaf water potential (Bucci et al., 2004; Meinzer et al., 2009). Such trees may have tight stomatal control, or isohydric regulation of water status, and larger hydraulic safety margins (McDowell et al., 2008). Thus, we expect low wood density species, particularly stem succulents, to have a high sensitivity to drought conditions and to be associated with acquisitive strategies.

Phenological patterns in SDTF exhibit a wide range of sensitives to rainfall and photoperiod (Borchert et al., 2002; Kushwaha et al., 2011) and may exert the dominant control on plant–water–carbon interactions (Vico et al., 2017). Still, we know little about the mechanisms behind phenological variations despite being under the same drought‐stress conditions. Phenological differences suggest that trees optimize temporal partitioning of water use according to functional group (Lasky et al., 2016) because it reflects ecophysiological functioning, including water use and access (Markesteijn et al., 2010; Valdez‐Hernández et al., 2010). In SDTF, the presence of evergreen species means either that they can access stable or deeper water sources or that they are highly specialized and efficient drought avoiders compared with the less drought tolerant deciduous species (Hasselquist et al., 2010; Jackson et al., 1995). While drought‐deciduousness is a well‐known avoidance strategy, a recent study reports that deciduous and evergreen trees had similar stomatal regulation patterns (de Souza et al., 2020), suggesting that, at best, phenology and other leaf traits are but rough simplifications that may not fully capture differences in hydraulic strategy (Powers et al., 2010).

The objective of this study was to test whether PFTs defined by wood density and phenology can be used to predict water use strategies in the Caatinga, a SDTF of northeast Brazil. Additionally, we compare species‐level differences to test and validate the PFT designation. We selected the Caatinga as a model biome for several reasons. First, because PFTs have been classified a prior for a large number of species (de Lima et al., 2012). Second, there is a high degree of biodiversity and endism (Zappi et al., 2015). Lastly, the Caatinga is at the more extreme end of aridity for SDTFs (Sampaio, 1995), so we expect a strong hydraulic response. We hypothesized the following divergent hydraulic strategies:

- Deciduous low wood density species (DL) are drought avoiders, lose leaves early in the dry season, are shallowly rooted, and have the lowest water use efficiency;
- Deciduous high wood density species (DH) have moderate drought tolerance, lose leaves late in the dry season, are shallowly rooted, and have moderate water use efficiency;
- Evergreen high wood density species (EH) are drought tolerant, deeply rooted and efficient water users.

We examined water use strategies with regard to (a) deciduousness and tolerance to water stress; (b) water use efficiency (WUE); and (c) water access and rooting depths. Tolerance to water stress was measured via predawn and mid‐day leaf water potential (Ψ_PD_ and Ψ_MD_), WUE was measured via the foliar δ^13^C, and water access was measured via soil and stem water δ^18^O and δ^2^H. This work is timely because our fundamental understanding of how SDTFs might respond to disturbances is limited. Specifically, evaluating the utility of the PFT framework is vital for modeling and predicting ecosystem resilience to changing climate (Pereira et al., 2020; Santos et al., 2014) and drought‐induced mortality (Powers et al., 2020). Such insight is urgent in the Caatinga, where drought frequency (Xu et al., 2019) and degradation and desertification are accelerating (Tomasella et al., 2018).

## 2 MATERIALS AND METHODS

### 2.1 Study site

**FIGURE 1:**
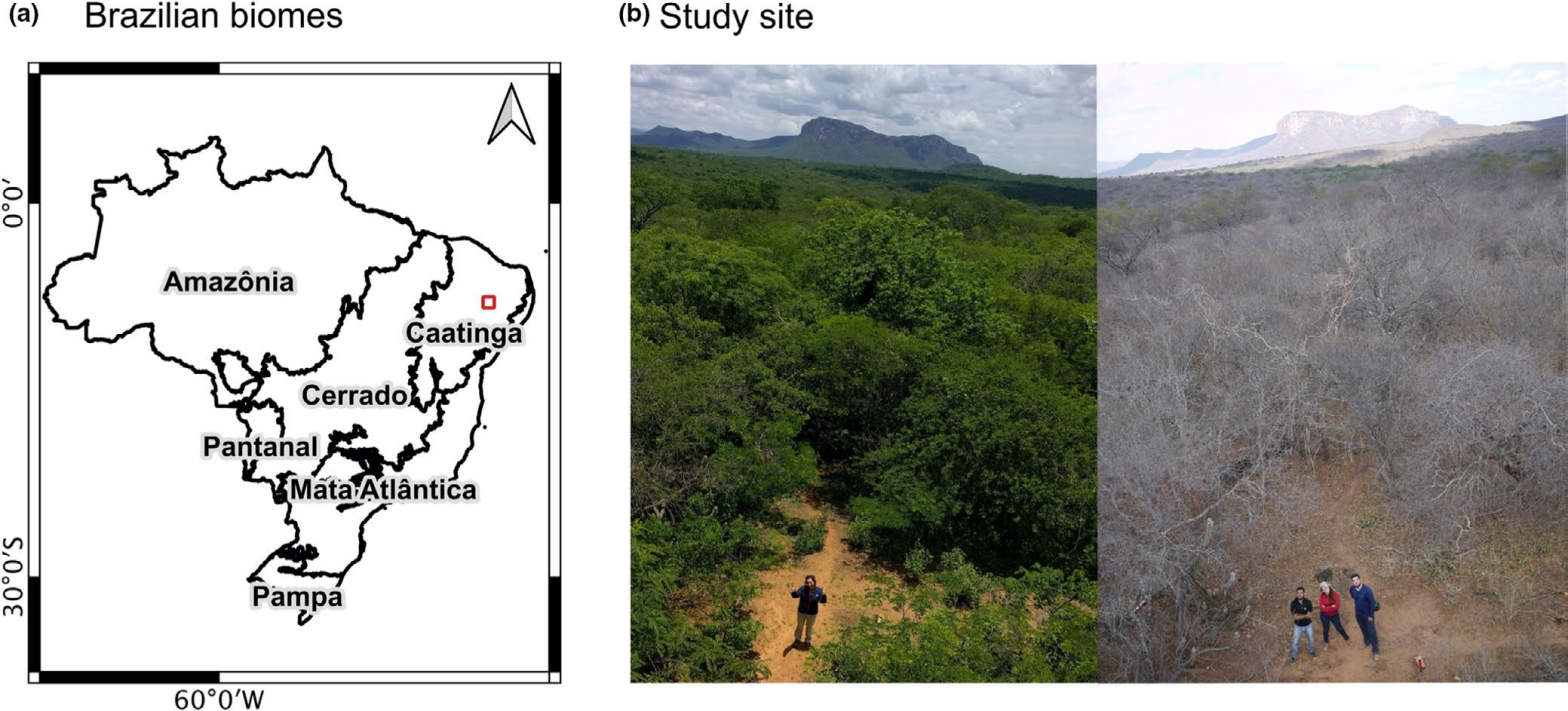
(a) Map of the different biomes within Brazil (adapted from IBGE, 2019) and (b) our study site in the Caatinga during the wet season (left; image take February 2018) and the dry season (right; image taken October 2018). Photo credit: C. Wright

Fieldwork was carried out within a relatively old (50+ years) forest stand, near the city of Serra Talhada, Pernambuco in the Caatinga biome of northeast Brazil (Figure 1a). The climate is tropical hot and dry (Koppen classification BShw). Recent annual rainfall (2000–2014) averaged 574 mm (Souza et al., 2016). The rainy season typically occurs from December to May and accounts for ~75% of annual rainfall. Average monthly air temperatures range from 21 to 26°C (Grieser et al., 2006, New LocClim 1.10). The study site (07°56′50″S and 38°23′29″W; mean elevation of c. 450 m) has drought‐deciduous vegetation (Figure 1b) and is rather unique in that few intact and contiguous stands of this age remain in the Caatinga (Antongiovanni et al., 2018). The landscape can be flat or hilly, and the lithology is crystalline (Moro et al., 2016). Soils are generally shallow, rocky and dominated by Neossolo Litolico and Luvissolo Crômico (Brazilian soil classification: Santos et al., 2014); or Entisol Orthent and Aridisol Argid (USDA soil classification: USDA, 1999).

### 2.2 Rainfall and soil water

Hydrometeorology is monitored at this site by the National Observatory of Water and Carbon Dynamics in the Caatinga Biome (https://ondacbc.eco.br/). This includes rainfall (TE525WS‐L rain gauge, Texas Electronics, Dallas, TX), air temperature, and humidity (HMP45C temperature and relative humidity probe, Vaisala, Campbell Scientific Inc., Logan, UT). Volumetric soil water content was monitored for one soil profile, at five depths: 5, 10, 20, 35, and 50 cm (5TM soil moisture sensor, Decagon Devices, Inc., Pullman, WA). Soil texture was characterized at four locations and five depths: 0–10, 10–20, 20–30, 30–40, and 40–50 cm (Table S1) and was used to scale soil water (minimum–maximum). Minimum or residual soil water content was inferred on the basis of USDA textural classification and Rosetta look‐up tables (Schaap et al., 2001; https://cals.arizona.edu/research/rosetta/). Maximum or saturated soil water content was based on measured soil properties. The monthly soil water storage was calculated by taking the daily difference in volumetric soil water content and aggregating across depth for each month.

### 2.3 Tree selection

We considered sixteen tree species belonging to three PFT classifications based on wood density and phenology (de Lima et al., 2012):

1. DL: *Amburana cearensis*, *Commiphora leptophloeos*, *Jatropha mollissima*, *Manihot epruinosa*, *Pseudobombax marginatum*, and *Spondias tuberosa*.
2. DH: *Anadenanthera colubrina*, *Aspidosperma pyrifolium*, *Bauhinia cheilantha*, *Cenostigma pyramidale*, *Croton blanchetianus*, *Enterolobium timbouva*, *Myracrodruon urundeuva*, and *Piptadenia stipulacea*.
3. EH: *Cynophalla flexuosa* and *Ziziphus joazeiro*

These species are representative of the Caatingas in Pernambuco (Rodal et al., 2008) and of SDTFs in general (Pereira et al., 2003; Särkinen et al., 2011). For each species, we selected three individuals, except for *S. tuerosa* and *C. flexuosa*, for which we only found two individuals (Table S2). In general, all individuals were of similar height (approximately 5 to 10 m), with the exception of *M. urundeua*, which can reach up to 20 m (Maia, 2012). PFT and species abbreviations are given in Table 1, and wood and leaf descriptions in Tables 2 and 3. The measurements performed on each individual are described below.

**TABLE 1:** Abbreviations and color coding for plant functional type (PFT) and species

PFT color and abbreviation	PFT	Species abbreviation	Species	Family
DL	Deciduous Low wood density	AMCE	*Amburana cearensis* (Allemao) A.C. Sm.	Fabaceae
		COLE	*Commiphora leptophloeos* (Mart.) Gillett	Burseraceae
		JAMO	*Jatropha mollissima* (Pohl) Baill.	Euphorbiaceae
		MAEP	*Manihot epruinosa* Pax & K. Hoffm.	Euphorbiaceae
		PSSP	*Pseudobombax marginatum* (A. St.‐Hil., Juss., & Cambess.) A. Robyns	Bombacaceae
		SPTU	*Spondias tuberosa* Arruda	Anacardiaceae
DH	Deciduous High wood density	ANCO	*Anadenanthera colubrina* (Vell.) Brenam	Fabaceae
		ASPY	*Aspidosperma pyrifolium* Mart.	Apocynaceae
		BACH	*Bauhinia cheilantha* (Bong.) Steud.	Fabaceae
		CEPY	*Cenostigma pyramidale* (Tul.) E. Gagnon & G. P. Lewis	Fabaceae
		CRBL	*Croton blanchetianus* Baill.	Euphorbiaceae
		ENSP	*Enterolobium timbouva* Mart.	Fabaceae
		MYUR	*Myracrodruon urundeuva* Allemão	Anacardiaceae
		PIST	*Piptadenia stipulacea* (Benth.) Ducke	Fabaceae
EH	Evergreen High wood density	CYFL	*Cynophalla flexuosa* (L.) J. Presl	Capparaceae
		ZIJO	*Ziziphus joazeiro* Mart.	Rhamnaceae

**TABLE 2:** The average and standard deviation (*SD*) of wood density (WD), diameter at breast height (DBH), and other characteristics for sixteen tree species of the Caatinga

PFT	Species	Plant form	WD, g/cm^3^ (*SD*)^a^			DBH, cm (*SD*)^a^				Trunk/bark characteristics^a^	Rooting characteristics^b^
DL	AMCE	Tree		0.44	(0.03)		7.4	(1.4)	Fleshy succulent; peeling bark; yellow to yellowish red cambia		Long fibrous fine roots and root tubers
	COLE	Tree		0.25	(0.06)		36.1	(22.3)	Fleshy succulent; peeling bark; green to greenish yellow cambia		–
	JAMO	Tree		0.26	(0.03)		4.1	(0.3)	Fleshy succulent pubescent; greenish yellow cambia		–
	MAEP	Tree		0.34	(0.04)		7.4	(–)	Slightly fleshy; scaly bark; dark gray cambia		Starchy tubers (wild cassava)
	PSSP	Tree		0.28	(0.05)		22.3	(–)	Smooth to ridged; green trunk		Swollen root
	SPTU	Tree		0.42	(0.03)		23.9	(–)	Thick scaly gray bark		Lateral roots and large tubers
DH	ANCO	Tree		0.63	(0.01)		11.6	(3.7)	Dark gray to dark brown bark; thick horn‐like spines		Shallow lateral roots
	ASPY	Tree		0.54	(0.02)		8.1	(0.2)	Light gray thin bark		–
	BACH	Shrub		0.61	(0.04)		3.5	(0.3)	Dark to light brown bark with slight peeling		Lateral roots
	CEPY	Tree		0.64	(0.02)		9.3	(4.7)	Light gray thin bark		Deep main root
	CRBL	Shrub		0.72	(0.01)		3.4	(1.1)	Dark to light brown bark with slight peeling		Lateral roots
	ENSP	Tree		0.61	(–)		10.3	(3.1)	Light gray thin bark		–
	MYUR	Tree		0.53	(0.02)		20.6	(2.6)	Thick scaly dark brown bark		Deeply rooted
	PIST	Tree		0.56	(0.05)		9.1	(–)	Light gray thin bark		–
EH	CYFL	Shrub / Liana		0.49	(0.05)		5.4	(–)	Smooth light gray trunk		–
	ZIJO	Tree		0.56	(0.02)		4.2	(1.8)	Light gray trunk; thorns		Deep radial roots

Note

See Table 2 for abbreviations and color code of plant functional type (PFT) and species.

^a^ Measured/observed.

^b^ Described inMaia (2012)

**TABLE 3:** Leaf characteristics for sixteen tree species of the Caatinga

PFT	ID	Leaf morphology^a^	Leaf texture^a^	Leaf flush	Leaf fall
DL	AMCE	Pinnately compound	Glabrous	Dry–wet transition^c^	Wet–dry transition^c^
	COLE	Pinnately compound	Pubescent	Dry–wet transition^c^	Wet–dry transition^c^
	JAMO	Palmately compound	Pubescent	Dry–wet transition^c^	Wet–dry transition^c^
	MAEP	Palmately compound	Glabrous	Dry–wet transition^c^	Wet–dry transition^c^
	PSSP	Palmately compound	Coriaceous, rugose	Wet season^b^	Wet–dry transition^b^
	SPTU	Pinnately compound	Glabrous	Dry–wet transition^b^	Dry season^b^
DH	ANCO	Bipinnately compound	Glabrous	Wet season^c^	Dry season^c^
	ASPY	Simple	Coriaceous	Wet season^c^	Dry season^c^
	BACH	Palmately compound	Pubescent	Wet season^b^	Dry season^b^
	CEPY	Bipinnately compound	Coriaceous, scabrose	Dry–wet transition^b^	Dry season^b^
	CRBL	Simple	Pubescent	Wet season^c^	Dry season^c^
	ENSP	Bipinnately compound	Coriaceous, scabrose	Wet season^a^	Dry season^a^
	MYUR	Pinnately compound	Pubescent	Wet season^c^	Wet–dry transition^c^
	PIST	Bipinnately compound	Glabrous	Wet season^c^	Dry season^c^
EH	CYFL	Simple	Coriaceous	Dry season^b^	Wet season^b^
	ZIJO	Simple	Glabrous, coriaceous	Wet season^b^	End of dry season^b^

Note

See Table 2 for abbreviations and color code of plant functional type (PFT) and species.

^a^ Observed.

^b^ Machado et al. (1997).

^c^ Lima and Rodal (2010).

### 2.4 Leaf water potential

To assess the tolerance to water stress, we measured leaf water potential using a Scholander pressure chamber (1505D, PMS Instrument Company, Albany, OR). Leaf water potential is an indicator of leaf hydration that is related to soil water content, stomatal closure, and hydraulic conductance (Tardieu & Simonneau, 1998; Martínez‐Vilalta & Garcia‐Forner, 2017). Measurements were made biweekly from 11 April to 22 August 2018, or as long as an individual had leaf cover (Table S2). Leaves collected at predawn (4:30–6:00 a.m.) and at mid‐day (11:30–1:00 p.m.) were clipped, bagged, and kept in a cooler with ice, then immediately measured in situ. The Scholander pressure chamber uses tanks of nitrogen gas within a closed system to apply a balancing pressure. Our tanks had sufficiently high pressure to make all but one of the measurements (Table S2; 665 total leaves).

Moreover, we defined changes in leaf water potential as ΔΨ = Ψ_MD_−Ψ_PD_ as an indicator of isohydric behavior. Here, a small ΔΨ is associated with isohydric behavior and a large ΔΨ is associated with as anisohydric behavior (Klein, 2014; Meinzer et al., 2016). Note however that isohydric behavior does not necessarily equate to tight stomatal control (Hochberg et al., 2018; Martínez‐Vilalta & Garcia‐Forner, 2017). Although isohydricity can be defined according to different metrics of leaf water potential yielding different interpretations, we retain this simple framework as a way to understand the daily fluctuations in the water potential in the context of the other measured traits.

### 2.5 Foliar δ^13^C

We collected samples for foliar δ^13^C analysis simultaneously as those for the leaf water potential measurements (Table S2). After collection, leaves were oven‐dried in the laboratory at 75 to 80°C for at least 48 hr, then transported to the Stable Isotopes for Biosphere Science (SIBS) Laboratory at Texas A&M University, College Station, for grinding, weighing, and packing. Once packed, the samples were analyzed for δ^13^C with an *Elemental Analyzer* (Costech Analytical Technologies, Valencia, CA) coupled to a *Thermo Scientific Delta V Isotope Ratio Mass Spectrometer (EA‐IRMS; Thermo Fisher Scientific, Waltham, MA)*. Stable isotope ratios are determined as the heavy to light isotope in the sample (R_sample_) or in the standard (Rs_tandard_) expressed in delta notation: δ (‰) = (R_sample_/R_standard_−1) × 1,000. The δ^13^C measurement was calibrated in accordance with certified standards: USGS (United States Geological Survey) Glutamic Acid 40 (δ^13^C = ‒26.39 ‰) and Glutamic Acid 41 (δ^13^C = 37.63‰). Quality control was performed in accordance with in‐house standards (δ^13^C = ‒12.78‰ and δ^13^C = ‒39.88‰). All δ^13^C measurements are reported on the scale V‐PDB scale.

At its simplest, foliar δ^13^C reflects the mole fraction of CO_2_ in the substomatal cavity, which is governed by stomatal regulation. When stomata begin to close, water loss decreases, and intercellular CO_2_ drops, reducing the apparent ^13^C fractionation. Thus, we broadly interpreted measured leaf C‐isotope ratios using the classical model which links variations in δ^13^C to variations in “intrinsic” water use efficiency (WUE, A/g_sw_; Farquhar et al., 1989; Farquhar & Lloyd, 1993; Seibt et al., 2008)—that is, the higher the foliar δ^13^C, the higher the WUE. However, differences in refixation of respired CO_2_ or mesophyll conductance have not been characterized for our species, with potential implications for our interpretation of observed variation in δ^13^C.

### 2.6 Stem water δ^18^O and δ^2^H

We used naturally occurring stable isotopes ratios of water (δ^18^O and δ^2^H) in stems, soils, and precipitation to trace the sources of water accessed by trees. A gradient of δ^18^O and δ^2^H can be observed across soil layers when the near‐surface soils become evaporatively enriched in the heavier isotopes, for example, ^18^O and ^2^H. This gradient is used to separate “shallow” versus “deeper” soil water sources when compared with the δ^18^O and δ^2^H in stems, assuming no fractionation upon uptake (Dawson & Ehleringer, 1991; Goldsmith et al., 2012).

Soil and stem samples representing wet conditions were collected on 10 April 2018, and those representing dry conditions were collected on 12 June 2018 from two to three individuals per species (Table S2). Samples for the EH group were collected separately on 19 September 2018. We collected the soil samples from seven pits at three integrated depths: 5–15 cm, 20–30 cm, and 40–50 cm. We could not obtain samples from the 40 to 50 cm depth for one pit in June and for two pits in September. Because the clay content is usually higher at these depths (Table S1), the soils shrank as they dried and became compacted and hard. Soils obtained from each integrated depth were placed into plastic bags so that they could be homogenized and subsampled. The stem samples were cut from individual trees. The bark was left on to avoid the influence of evaporation, although this potentially might introduce a small error from phloem water (Ellsworth & Williams, 2007). Both soil and stem samples were placed in glass vials and stored in a cooler with ice in the field.

Rainwater and throughfall samples were collected approximately biweekly from 18 January 2018 to 9 June 2018, in collectors wrapped with aluminum foil filled with a thin layer of paraffin oil to reduce evaporation (IAEA, 2014). We also collected groundwater and surface water samples from the surrounding area. Water samples containing debris were filtered (0.2 μm) to remove particles.

All the soil and stem samples were frozen, and the water samples were refrigerated, until analysis at the SIBS Laboratory. Water was extracted from stems and soils via cryogenic vacuum distillation (West et al., 2006) a bath of boiling water (100°C). The extraction was considered complete when no more water condensed, and no more water vapor could be seen on the liquid nitrogen side of the line. Although extraction temperature and other variables (e.g., soil texture and mineralogy) potentially interact to affect the isotopic composition of extracted water (Adams et al., 2020), we chose to perform extractions at 100°C to be consistent with other published work and because soil clay content likely did not change much within our sampling area (Orlowski et al., 2016).

All samples were analyzed using a High Temperature Conversion/Elemental Analyzer coupled to a Delta V Advantage Isotope Ratio Mass Spectrometer (TC/EA‐IRMS). Stable isotopic composition is expressed in delta notation (δ). The water isotope ratios measurements were calibrated in accordance with in‐house water standards: SIBS‐wA (δ^2^H = −390.8 ± 1.6‰, δ^18^O = −50.09 ± 0.33‰) and SIBS‐wP (δ^2^H = −34.1 ± 1.9‰, δ^18^O = −4.60 ± 0.24‰). Quality control was performed in accordance with an in‐house water standard, SIBS‐wU (δ^2^H = −120.2 ± 1.5‰, δ^18^O = −15.95 ± 0.27‰). These in‐house standards were established on the basis of IAEA reference waters (VSMOW2, SLAP, and GISP). All water isotope ratios are reported on the V‐SMOW scale.

Additionally, we calculated the shift in stem water isotopic composition as Δδ^18^O = δ^18^O_T2_−δ^18^O_T1_. For each PFTs, we applied this calculation as δ^18^O_June_−δ^18^O_April_ to account for drying soils. We also applied this calculation as δ^18^O_Sept_−δ^18^O_June_ for the EV species only, since these were the only trees which still had leaf cover in September. A similar calculation was performed for Δδ^2^H.

### 2.7 Wood density

We collected branch samples from three individuals per species to measure the stem‐specific wood density. We collected samples from only one *Enterolobium timbouva* individual and only two *Cynophalla flexuosa* individuals because of limited occurrence. We used the water‐displacement method (according to Pérez‐Harguindeguy et al., 2013) to find the volume of saturated wood samples. The samples were then oven‐dried at 105°C for at least 72 hr, and the dried mass was reweighed to calculate wood density (Table 2).

### 2.8 Statistical analysis

To test whether PFT predicts species’ water use strategies, we conducted repeated ANOVA tests using linear mixed‐effects modeling in the R package nlme (Pinheiro et al., 2021). The response traits we modeled were Ψ_PD_, Ψ_MD_, ΔΨ; foliar δ^13^C; and stem water δ^18^O and δ^2^H. To determine for significance, we applied Tukey's post hoc test for linear mixed models using the R package multcomp (Hothorn et al., 2008). First, we modeled PFT as the fixed effects factor, with date and species as the random effects factors (i.e., repeated measures). Species was included as a random effects factor since we could have selected other representative species. Second, to test the correspondence of each species to its assigned PFT, we modeled species as a fixed effects factor within each PFT and date and species as the random effects factors. We did not model stem water δ^18^O nor δ^2^H at the species level because the sample size was too small (Table S2). For all models, we confirmed homogeneity in the variance of the residuals.

## 3 RESULTS

### 3.1 Rainfall and soil water

**FIGURE 2:**
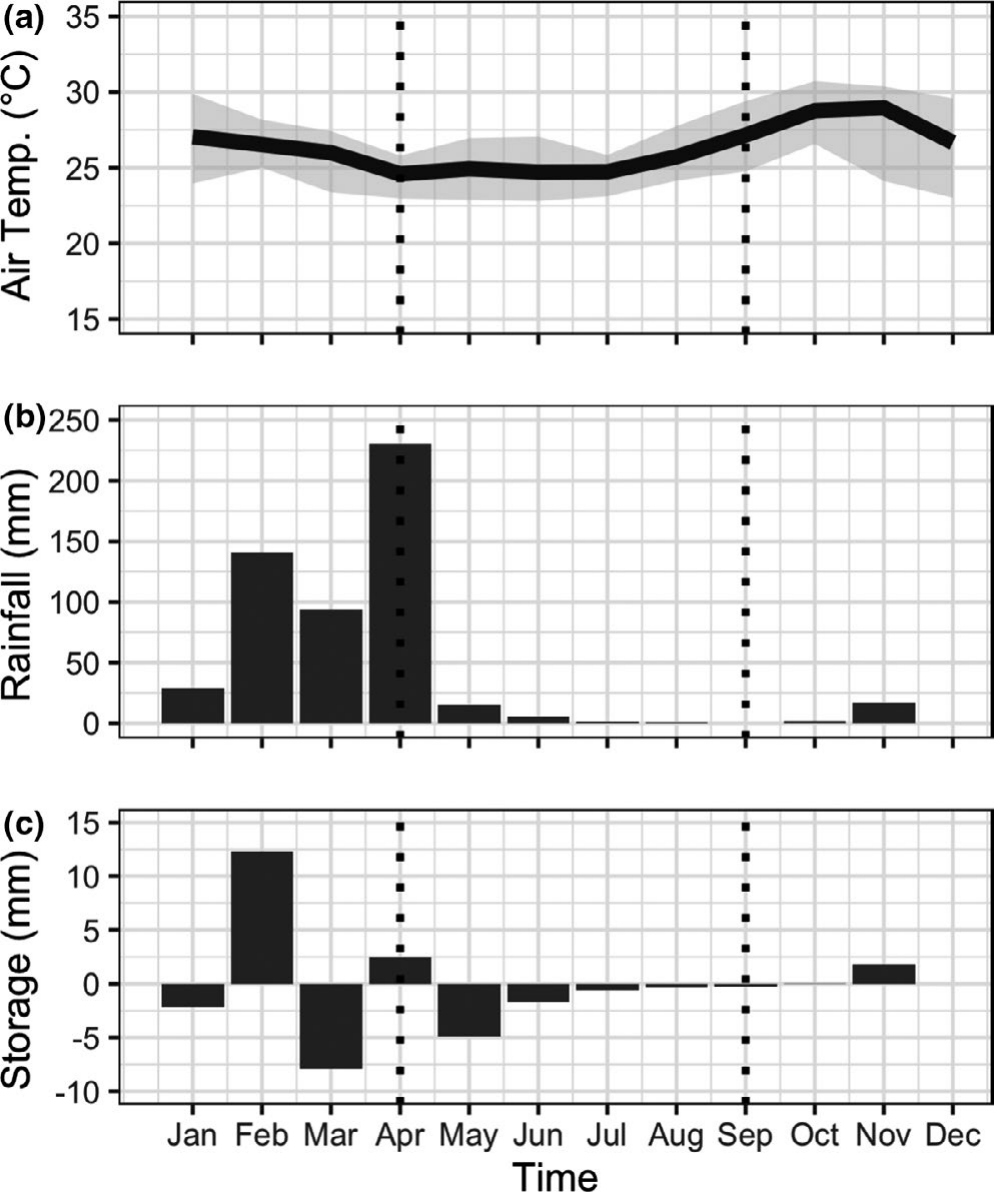
(a) Average monthly air temperature (gray shading denotes minimum and maximum bounds); (b) total monthly rainfall; and (c) monthly soil water storage for the top 50 cm. The vertical dashed lines demarcate the sample collection period (10 April–22 August 2018)

The start of the rainy season in 2017 was observed with the first large event pulses on December 24 (24 mm was registered in the city of Serra Talhada and 10 mm at the Cachoeira weir, the nearest station to the study site—http://www.apac.pe.gov.br). The end of the rainy season was at the end of April 2018, which was the wettest month of the study period with a total rainfall of 230 mm. We did not observe large rain events in May, when monthly rainfall dropped to 15 mm. Average monthly temperatures increased from 24.6°C in April to 25.7°C in August. The lowest daily temperature was 22.8°C, and the highest daily temperature was 27.8°C. Soil water storage was positive during the months of February and April (12.3 mm and 2.4 mm). Soil water storage was also positive in November (1.9 mm), which would correspond to the following wet season (Figure 2). Regarding our stem and soil sample collection, the “wet” period collection on April 10 took place the day after a large rain, registered as a 77 mm event; the “dry” period collection on June 12 took place after 40 days without any rain events larger than 5 mm and during which soil water storage was negative; and additional “dry” period collection for EH only took place on September 19 to 20.

### 3.2 Leaf water potential

**FIGURE 3:**
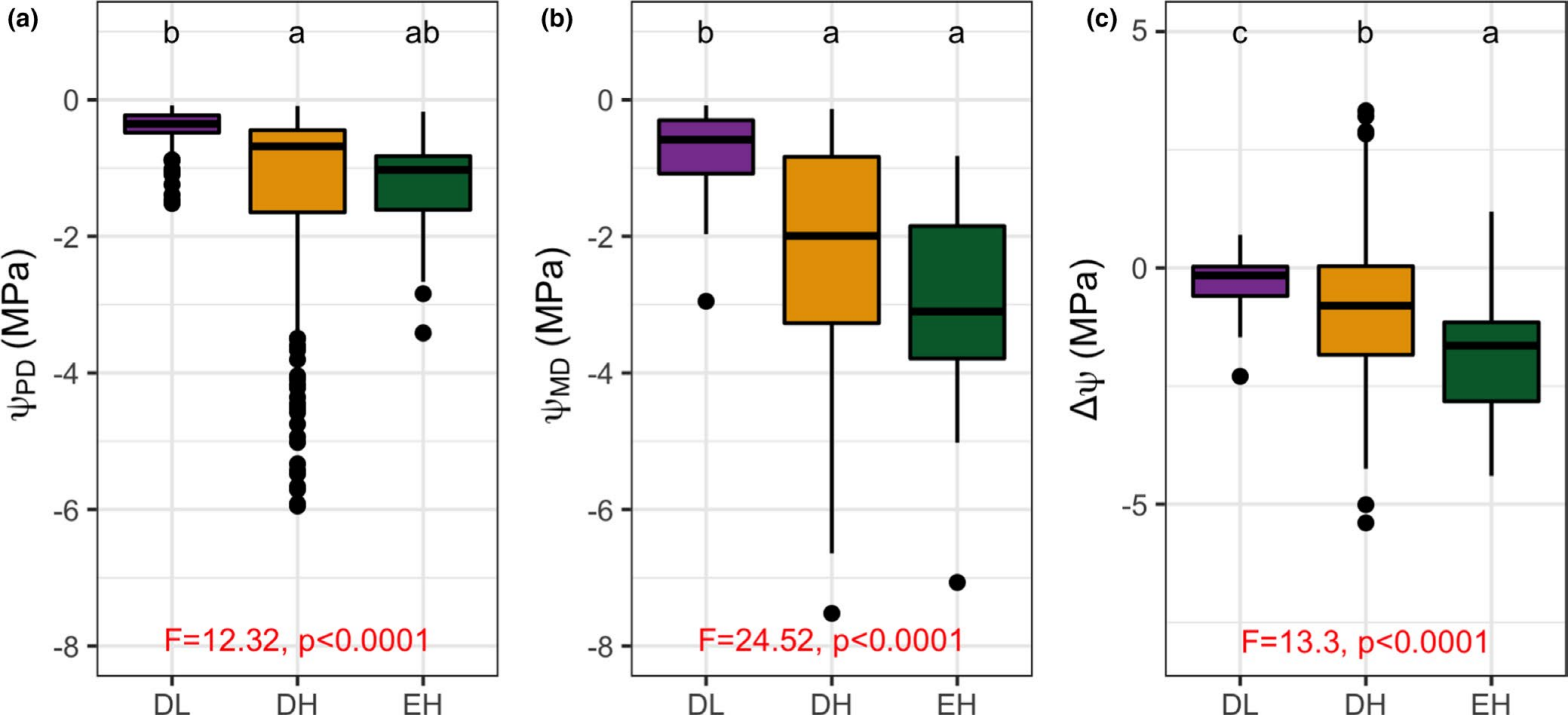
Leaf water potentials for the three PFTs at (a) predawn (Ψ_PD_), (b) mid‐day (Ψ_MD_), and (c) the difference between them (ΔΨ), measured biweekly between 11 April and 22 August 2018. Error bars are standard deviations. PFTs are color‐coded. Significant linear mixed‐effects model results are denoted by the F statistics and p‐values shown in red. Letters denote pair‐wise comparisons from Tukey's post hoc procedure. See Table 2 for PFT color code

Leaf water potential results differed by PFT (Figure 3). There were significant differences between DL and DH species with respect to both Ψ_PD_ and Ψ_MD_, but there were no significant differences between DH and EH with respect to Ψ_MD_. All three PFTS differed significantly with respect to ΔΨ. Trends over time differed for DL compared with DH and EH (Figure S3). For DL, Ψ_PD_ and Ψ_MD_ was high and relatively stable even as soils dried; Ψ_PD_ remained above −1 MPa. For DH, Ψ_PD_ quickly decreased, but for EH, Ψ_PD_ decreased more gradually. Similar trends were observed for Ψ_MD_ values. For DH, Ψ_MD_ fell gradually from an average of just above −1 MPa in April to an average of almost −5 MPa in July. For EH, Ψ_MD_ was variable over time; values began at an average of less than −1 MPa in April and reached just below −4 MPa in July.

**FIGURE 4:**
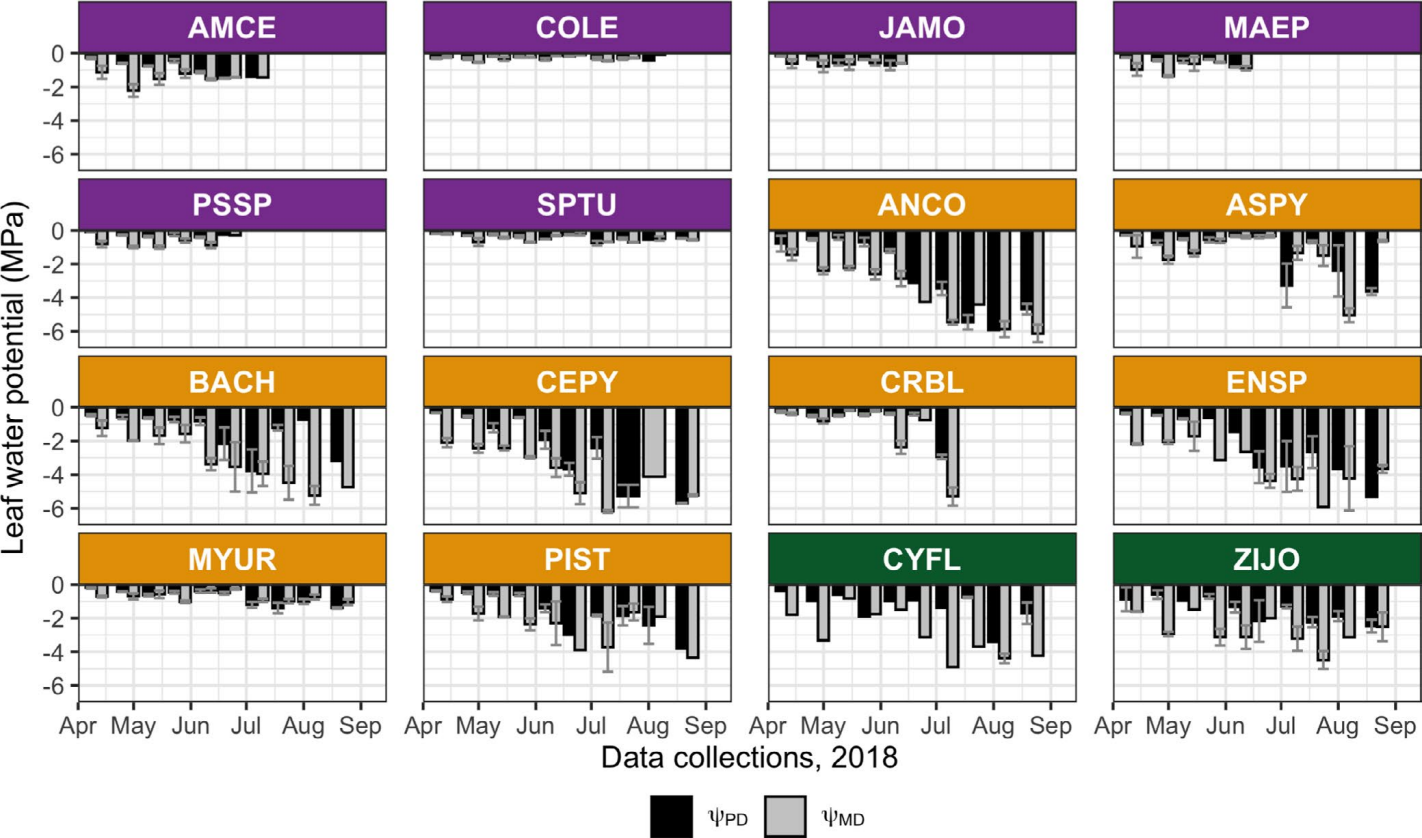
Species‐level leaf water potentials measured biweekly from 11 April to 22 August 2018. Error bars are standard deviations. See Table 2 for PFT color code and species abbreviations

Temporal patterns at the species level (Figure 4) reflect phenological differences. The DL species lost all leaf cover early in the dry season, shortening the measurement period. Exceptions were *Commiphora leptophloeos* and *Spondias tuberosa*, which retained some leaf cover into early August. These two species had higher Ψ_PD_ and Ψ_MD_ than the other DL species (Table S3) and were more similar to each other in Ψ_MD_ and ΔΨ than to the other species in the group. All DL species had very similar Ψ_PD_ values (Figure 4; Table S3).

Most of the DH species showed similar values and trends to each other (Figure 4; Figure S2). Both Ψ_PD_ and Ψ_MD_ declined as the dry season progressed. Exceptions were *Aspidosperma pyrifolium*, *Croton blanchetianus*, and *Myracrodruon urundeuva*. *A. pyrifolium* and *C. blanchetianus* had relatively stable Ψ_PD_ and Ψ_MD_ from April to June, but then changed abruptly as they lost all leaf cover. For *A. pyrifolium*, Ψ_PD_ and Ψ_MD_ remained above −2 MPa before dropping to −3 MPa in July and −5 MPa in August. For *C. blanchetianus*, Ψ_PD_ and Ψ_MD_ remained close to −1 MPa—except for June 9 and July 7, when Ψ_MD_ fell substantially (~ −2 MPa and −5 MPa, respectively). The ΔΨ (Figure S2) showed that *A. pyrifolium* differed significantly from only two other species in the group, and *C. blanchetianus* did not differ significantly from any of them. The behavior of *M. urundeuva* appeared more similar to that of the DL group, remaining higher and stable through the dry season. The Ψ_MD_ of *M. urundeuva* was significantly different from that of five other DH species (*p* < .0001), although its ΔΨ values did not differ significantly (Figure 7).

The species within the EH group appear to have similar Ψ_PD_ and Ψ_MD_ trends (Figure 4) but which are not predicted by date (Figure S2; *F* = 0.05, 0; *p* = .84, .97). For most of these species, Ψ_PD_ and Ψ_MD_ dropped as the dry season progressed.

### 3.3 Foliar δ^13^C

**FIGURE 5:**
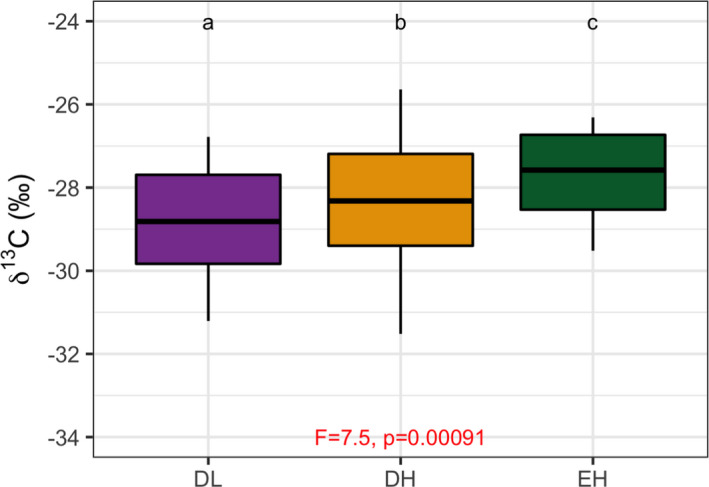
Averaged foliar δ^13^C by PFT for the period 28 April to 22 August 2018. Error bars are standard deviations. Significant linear mixed‐effects model results are denoted by the F statistics and p‐values shown in red. Letters denote pair‐wise comparisons from Tukey's post hoc procedure. See Table 2 for PFT color code

The foliar δ^13^C differed significantly by PFT, with DL showing the lowest values, followed by DH and then by EH (Figure 5; *F* = 7.5, *p* = .001). Over time, both DL and EH decreased (from −28.9‰ to −29.2‰ and −27.5‰ to 27.8‰, respectively; Figure S3), whereas DH generally increased (from −28.5‰, to −28.0‰; Figure S3).

**FIGURE 6:**
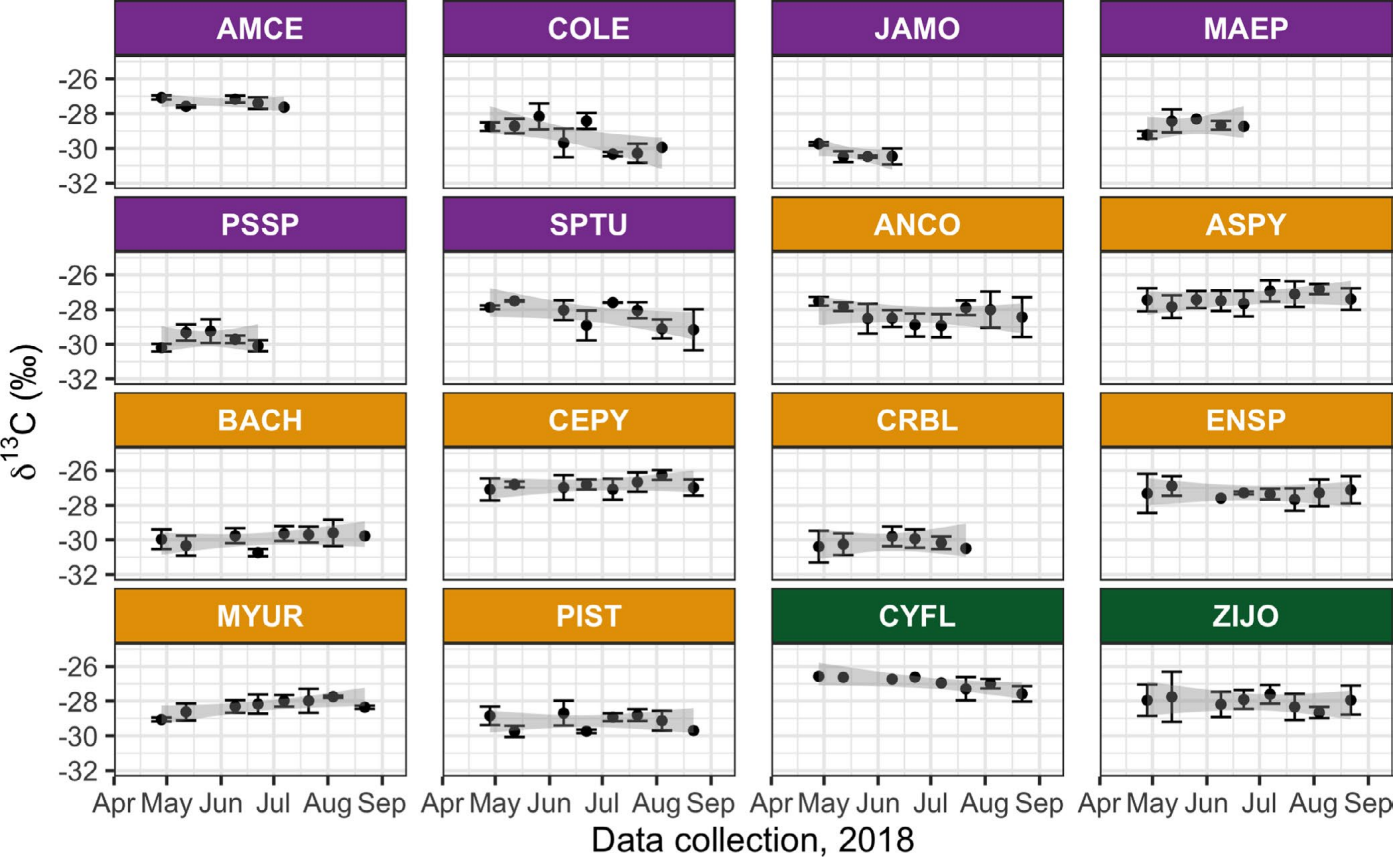
Trends in species‐level foliar δ^13^C for the period 28 April to 22 August 2018. Error bars are the stand deviations per sampling date. See Table 2 for PFT color code and species abbreviations. Gray shading depicts the standard deviation associated with the linear regression fit

At the species level (Figure 6), trends in foliar δ^13^C remained mostly stable, as expected. Notably *Myracrodruon urundeuva* increased slightly, and *Commiphora leptophloeos*, *Cynophalla flexuosa*, and *Spondias tuberosa* decreased slightly. Species that tended to have higher foliar δ^13^C (above −28‰) were *Amburana cearensis*, *C. flexuosa*, *Aspidosperma pyrifolium*, *Cenostigma pyramidale*, and *Enterolobium timbouva*—several of these belonging to the Fabaceae family (Table 1). The highest average foliar δ^13^C was found for *A. pyrifolium* (Table S3; −27.34‰ ± 0.94‰). Species with lower ratios, close to −30‰, were *Jatropha mollissima* and *Pseudobombax marginatum* in the DL group, and *Bauhinia cheilantha* and *Croton blanchetianus* in the DH group. Moreover, there was significant species‐level variation in average foliar δ^13^C within each PFT (Figure S4). Among DL species, *A. cearensis* and *J. mollissima* were the most different. There was quite a bit of variation between species in the DH group. The two EH species, *C. flexuosa* and *Ziziphus joazeiro*, were significantly different from each other.

### 3.4 Stem water δ^18^O and δ^2^H

**FIGURE 7:**
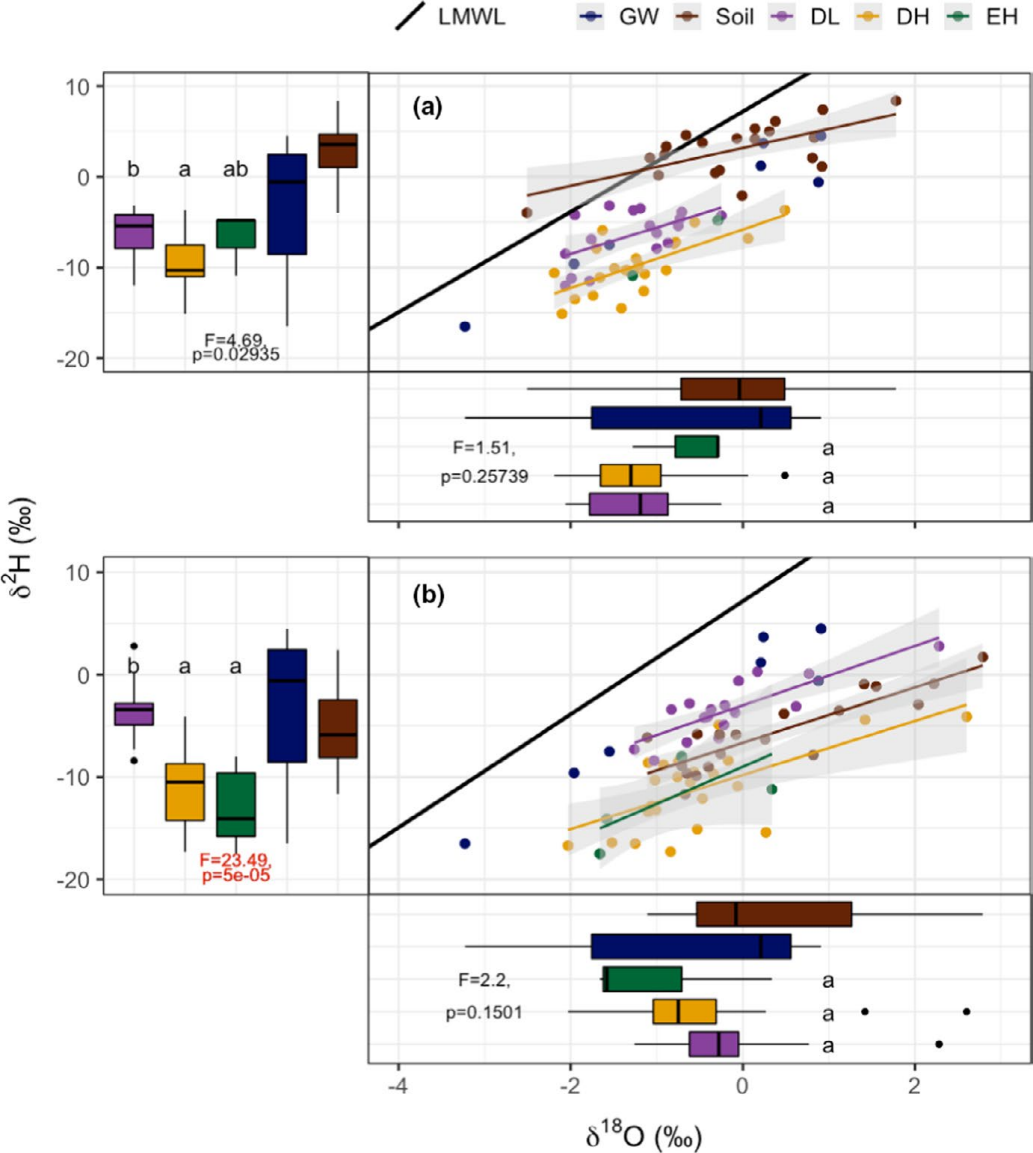
Dual‐isotope plots by PFT for (a) 10 April 2018 during the wet season and (b) 12 June 2018 during the dry season. The local meteoric water line (LMWL; slope = 5.53; intercept = 7.19) was calculated using rain samples collected from December 2017 to June 2018. Boxplots show the average (line) and standard deviation (error bars) in δ^18^O and δ^2^H for ground water (GW), soil water, and PFT stem water. Linear mixed‐effects model results for PFTs are denoted by the F statistics and p‐values, with significant differences in red. Letters denote statistical grouping‐based Tukey's post hoc. See Table 2 for PFT color code and species abbreviations

There is a separation between PFTs in dual isotope space, driven by differences in δ^2^H but not δ^18^O (Figure 7). During the wet season (Figure 7a), both DL and DH plot below the soil evaporation line but are captured by rainfall and groundwater values; the EH regression line is not shown because only two stem samples were collected. During the dry season (Figure 7b), DL lies above the soil evaporation line, whereas DH and EH lie below.

Results based on δ^18^O differed from those based on δ^2^H. With respect to stem water δ^18^O, PFTs were not significantly different for either sampling date (Figure 7a: *F* = 1.51, *p* = .257 for April; Figure 7b: *F* = 2.2, *p* = .150 for June), but they were with respect to stem water δ^2^H (Figure 7a: *F* = 4.69, *p* = .02935 for April; Figure 7b: *F* = 23.49, *p* = .00005 for June). In April, δ^2^H for DL was significantly different from EH, but not from DH. In June, DL was significantly different from both DH and EH. The difference between DL and DH species is apparent only when based on δ^2^H. For potential source water, groundwater and soil water had similar ranges which encompassed the isotopic ratios of all PFTs.

**FIGURE 8:**
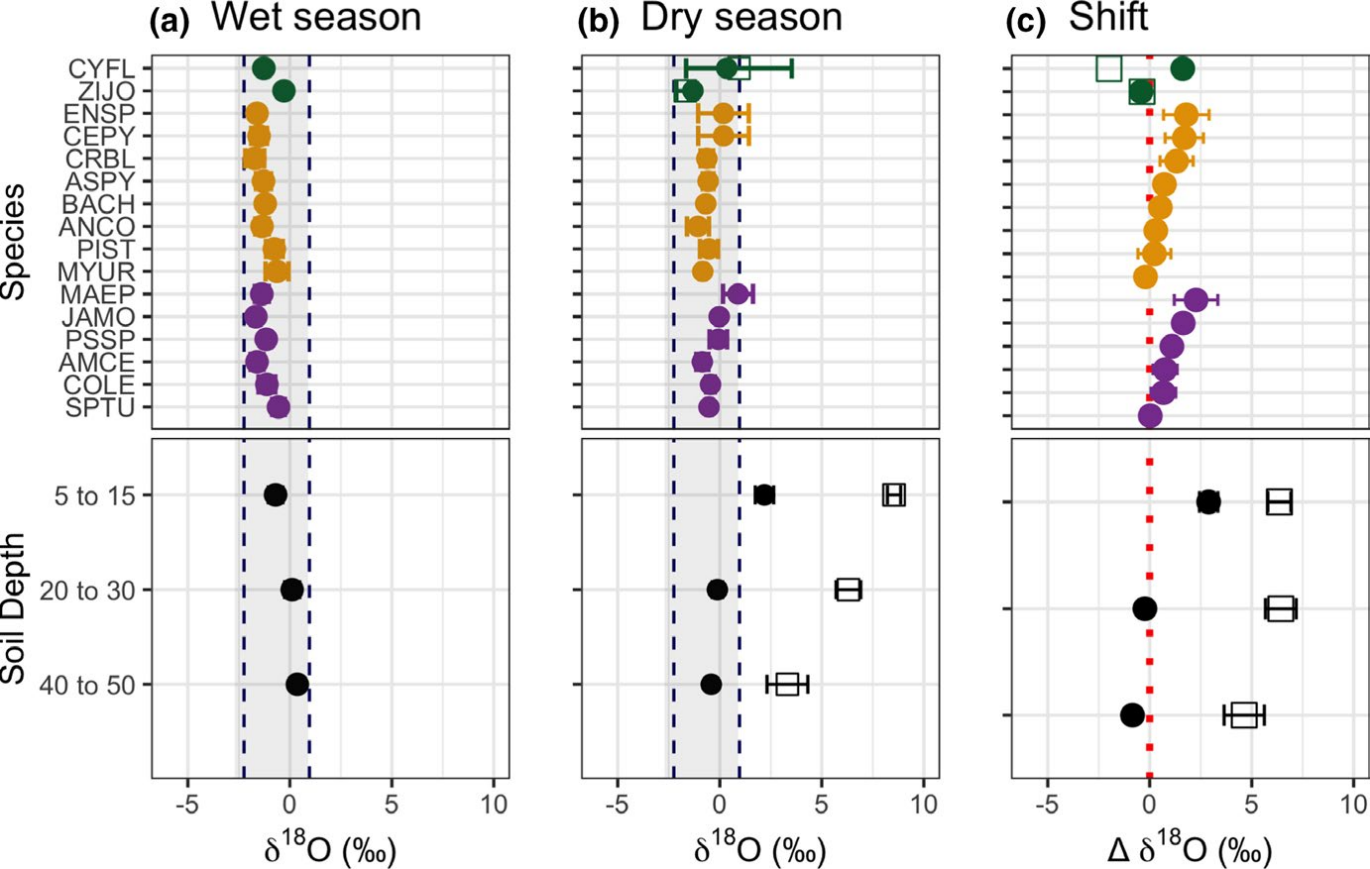
Stem and soil water δ^18^O for (a) the wet season (solid point: 10 April 2018); (b) the dry season (solid point: 12 June 2018; empty square: 19 September 2018 for EH only); and (c) the shift between sampling dates, Δ δ^18^O (solid point: difference between June and April; empty square: difference between Sept. and June for EH only). For reference, the average ± standard deviation of the rainwater (gray shading) and groundwater (dashed lines) are also shown. See Table 2 for PFT color code and species abbreviations

Shifts in isotopic ratios at the PFT level show a difference in Δδ^18^O between DL and EH, although this was not significant (Figure S5: *F* = 3.35, *p* = .067). At the species level, most species experienced isotopic enrichment, particularly in terms of Δδ^18^O (Figure 8). Only *M. urundeuva*, a DH species, and the two EH species, shifted to heavier δ^18^O into the dry season. For *C. flexuosa*, stem water first shifted toward heavier δ^18^O from April to June, and then toward lighter δ^18^O from June to September, although this calculation is based on only one individual. The other EH species, *Z. joazeiro*, showed a consistent shift to lighter δ^18^O. A slightly different trend was observed in terms of Δδ^2^H (Figure S6). All DL species shifted toward lighter δ^2^H, but most DH and EH species shifted toward heavy δ^2^H.

## 4 DISCUSSION

### 4.1 Why does leaf water potential differ by PFT?

We found that leaf water potential, along with wood density, can be consistently used to define PFTs in this biome. This agrees other studies in the Caatinga (de Lima et al., 2012; Oliveira et al., 2015), Costa Rica (Borchert, 1994; Hoeber et al., 2014), and Mexico (Valdez‐Hernández et al., 2010). Our study expands on previous work in that we tracked both Ψ_PD_ and Ψ_MD_ at a higher temporal frequency to better understand the interaction between tree responses and functional traits, such as leaf water potential and wood density, with local environmental cues (Feng et al., 2019).

We found that DL species had consistently high Ψ_PD_ regardless of season, in contrast to DH and EH species, which displayed lower and decreasing Ψ_PD_ into the dry season and were not significantly different from each other. This distinction indicates either that (a) DL have a more reliable water source, if Ψ_PD_ reflects soil water availability, or (b) that leaf water status eventually decouples from the atmosphere and/or soil as it dries. It is unlikely that difference in water source, brought about either by variation in soil or rooting depth, is the cause of high Ψ_PD_. The use of deep soil water is not supported by stem δ^18^O nor δ^2^H. Rather, DL species likely rely solely on shallow soil water resources particularly in the form of rainfall pulses wetting near‐surface soils. Thus, it is more probable that leaf water status decouples from the soil in a process known as “hydraulic fuse”. This notion discussed in more detail below and as related to Ψ_MD_.

We also found that for DL species, Ψ_MD_ was higher and ΔΨ was smaller than for the other two PFTs. Our results suggest that DL species with significant water storage regulate stomata as a response to water stress (during mid‐day hours), thereby reducing water loss, protecting larger, more vulnerable xylem vessels, while maintaining C uptake. As species approach critical levels of Ψ_MD_—which could lead to cavitation, embolism, and ultimately hydraulic failure—early leaf shedding by these species acts as a hydraulic fuse: It decouples trees from the atmospheric demand, slows water loss, and stabilizes leaf water potential (Wolfe et al., 2016). This type of early leaf‐shedding behavior for low wood density species, even under moderate stress, has been previously documented in the Caatinga (de Lima et al., 2012; Lima et al., 2018), in other SDTFs with stem succulent trees (Borchert et al., 2002; Worbes et al., 2013), and even in flood‐pulsed tropical forests (Schöngart et al., 2002). For species having a particularly large leaf area, such as *J*. *mollissima*, *M. epruinosa*, and *P. marginatum*, we observed that all leaves were lost relatively quickly. Additionally, the fact that DL species show high Ψ_PD_ and Ψ_MD_ suggests that early shedding of fine roots, particularly if shallow, also occurs. Here, the air gap formed between roots and soils would act as the hydraulic fuse decoupling plant and soil water potentials (Cuneo et al., 2016). This hypothesis should be tested in future work on this system.

Of the DL species, we investigated, *C. leptophloeos* and *S*. *tuberosa* retained some (much reduced) leaf cover into the dry season—probably because they have greater water storage capacity. *C*. *leptophloeos* has the lowest wood density, greatest diameter, and therefore the greatest stem water storage capacity. *S*. *tuberosa* likely has the greatest root water storage capacity, given the presences of a significant number and size of xylopodia (Antunes et al., 2016; Filho, 2001). Xylopodia are large root tubers that store water and carbohydrates. Moreover, they can help maintain turgor by modifying solute levels via osmotic adjustment (Silva et al., 2011). Numbering from hundreds to over a thousand per tree and weighing an average of 1.78 kg (Cavalcanti & Resende, 2006), these can hold significant reserves of water, which seems to be important for DL species that flush or flower at the end of the dry season and before the start of the first rains (Chapotin et al., 2006; de Lima et al., 2012). Whether xylopodia are present in other DL is not well documented.

In contrast, the DH and EH groups both had lower Ψ_PD_ and Ψ_MD_ values and larger ΔΨ, particularly as soils dried. These two PFTs are able to tolerate significant soil moisture limitations because of their more resistant hydraulic architecture (smaller vessel diameters and stronger vessel walls). This anisohydric behavior (larger ΔΨ) indicates that trees in these two PFTs are able to exploit soil water held under higher tension. Success in meeting water demands is evident in their phenology. When these trees can no longer obtain adequate moisture from the soil, they lose their leaves. Thus, the timing of leaf fall varies not only according to soil water availability but also the species’ ability to tolerate low water potentials (Lima & Rodal, 2010). That EH species are able to maintain leaf cover year‐round means that, compared with DH species, they either are better at taking up limited soil water resources or are more efficient in using available water. Indeed, EH species had the greatest WUE and was the only group to have shifted to potentially alternative water sources.

Intriguing exceptions to the general DH trends were observed for *A. pyrifolium*, *C. blanchetianus*, and *M. urundeuva*. For the first two, Ψ_PD_ and Ψ_MD_ were higher during the early part of the dry season, then began declining after July. According to our field observations, *C. blanchetianus*, a species characterized by fine pubescent leaves, soon lost all leaf cover; and *A. pyrifolium*, a species characterized by thick, waxy coriaceous leaves that may help minimize water loss during high water‐stress conditions (Medeiros et al., 2017; Oliveira et al., 2003), retained a diminished level of leaf cover into the middle of the dry season and had relatively more efficient water use. Some have reported that under favorable conditions *A. pyrifolium* will keep leaf cover year‐round (Machado et al., 1997). In the case of *M. urundeuva*, it is unclear how this species maintained high and stable Ψ_PD_ and Ψ_MD_; we also found a relatively low WUE. Potential explanations for these observations are related to water storage and source. For example, young *M. urundeuva* have a well‐developed taproot that is thought to store water (de Figueirôa et al., 2004; Maia, 2012), but whether these water‐storing root vessels persist into maturity is not known but could explain high leaf water potentials and the lack of change in stem water δ^18^O. Further, this species has pinnately compound pubescent leaves, which may help reduce foliar water loss to some degree.

### 4.2 Why is there substantial variation in species‐level foliar δ^13^C?

Foliar δ^13^C differed significantly by PFT. In addition, we found substantial variations at the species level, masked by the PFT‐level aggregation. Different species can exhibit a wide range of foliar δ^13^C because it is related to stomatal conductance, altered C‐to‐N allocation to carboxylation, and leaf structure (Seibt et al., 2008).

We found that DL species had the lowest foliar δ^13^C, indicating that as a group, these trees are the least efficient water users. This result is consistent with the idea that DL are “fast‐growing,” and favor rapid C assimilation over efficient water use (Reich, 2014). In an SDTF in Costa Rica, Worbes et al. (2013) found that stem succulent species showed higher foliar δ^13^C than did deciduous, brevi‐deciduous, and evergreen species because of a mid‐day reduction in stomatal conductance.

A closer look at foliar δ^13^C over time for the DL group reveals a decreasing trend driven by *C. leptophloeos* and *S*. *tuberosa* (the other DL species had mostly constant foliar δ^13^C). This decreasing trend may, in part, reflect the refixation of plant‐respired CO_2_ and the influence of corticular photosynthesis (Cernusak & Hutley, 2011; Pfanz et al., 2002). Because respired CO_2_ is high in ^12^C, similar to plant tissue, its refixation would have the effect of reducing foliar δ^13^C. It is also plausible that corticular photosynthesis occurs, as has been found for other woody species with green stems, including several from the Anacardiaceae, Burseraceae, Euphorbiaceae, and Fabaceae family (Ávila‐Lovera et al., 2020). To our knowledge, this has not been explicitly verified for any of the species in this study which have a greenish cambium, such *C. leptophloeos*, *J. mollissima*, and *P. marginatum*. Another factor that could reduce foliar δ^13^C is the remobilization and reallocation of nutrients (Munné‐Bosch & Alegre, 2004), and potentially of carbon, from older to younger leaves as the older leaves senesce before abscission. For example, *S*. *tuberosa* displayed strong senescence, its leaves turning from green to yellow to red. Additionally, *C. leptophloeos* and *S*. *tuberosa* showed the least change in stem water isotope ratios—meaning that water stored in tree tissue during the wet season was an important reserve. Analysis of root water samples collected from the xylopodia of two *S*. *tuberosa* individuals in late September indeed revealed δ^2^H and δ^18^O similar to those of wet season soil water (S2).

For the DH species, foliar δ^13^C was intermediate and showed a slight increase over time, but species‐level trends were mainly constant. For species such as *A. pyrifolium*, *C. pyramidale*, and *Enterolobium timbouva*, higher foliar δ^13^C could be related to leaf traits. These species tended to have smaller leaf areas, greater leaf thickness, and a waxy epicuticular coating (e Silva et al., 2014; Medeiros et al., 2017; Oliveira et al., 2003). The EH species had the highest foliar δ^13^C, indicating that this PFT was the most efficient water user. High foliar δ^13^C can occur when stomatal closure reduces water loss and promotes the use of internal leaf CO_2_. High foliar δ^13^C is consistent with the fact that species like *C. flexuosa* have small, coriaceous leaves, which minimize water loss. Other research did not find differences in intrinsic WUE between deciduous and evergreen species in the Caatinga (de Souza et al., 2015), but this study did not take into account prominent differences in wood density.

### 4.3 Why is vertical root partitioning of soil water sources limited?

The stem water δ^18^O and δ^2^H results indicate trees in the Caatinga depend largely on shallow soil water. Although we analyzed both, we rely more heavily on interpretations based on δ^18^O. First, δ^2^H is less sensitive to kinetic fractionation effects experienced in evaporating soils, making it a slightly less useful tracer of soil water sources (Mathieu & Bariac, 1996). Second, fractionation between xylem sap and stem tissue water may occur for δ^2^H but not for δ^18^O (Zhao et al., 2016). This could be why our stem water samples yielded lighter δ^2^H for the DH group even during the wet season. Barbeta et al. (2020) showed that stem water δ^2^H became significantly lighter when soil water dropped below the permanent wilting point, and when trees experienced significant declines in stomatal conductance and Ψ_PD_— conditions which occur during the dry season at our study site, and which would mainly affect DH and EH species. Therefore, the change toward lighter δ^2^H values that we observed for the DH group could have been altered by fractionation processes; something that does not appear for δ^18^O, and in which DL and DH species had similar values. Lastly, because precipitation, groundwater, and soil water had similar ranges which encompassed the isotopic ratios of all PFTs, statistical analysis of source water remains inconclusive. For these reasons, we rely on δ^18^O to assess relative differences in rooting depth, although we acknowledge that potentially important uncertainties remain in the use of water isotopes to infer rooting depth.

If there is vertical partitioning between PFTs, the relative difference is strongest between DL versus EH when soils are dry and both groups are still transpiring (i.e., in flush), with DH occupying the intermediate isotope space. Still, an important consideration is that the soils at our study site and, in much of the crystalline basement region of the Caatinga, are shallow or rocky (Moro et al., 2016; Santos et al., 2012). These soils rarely exceeding 60 cm in depth (Albuquerque et al., 2015) and have an effective tree‐rooting depth of 40 cm (Pinheiro et al., 2013). Recent studies on DH species found that root biomass was largely concentrated near the tree base and within the top 20 to 40 cm of soil (Albuquerque et al., 2015; Costa et al., 2014). The growth of fine roots is driven by water availability, which explains why there is high investment in root length per unit of biomass in surface soils (Andrade et al., 2020)—roots must maximize lateral exploitation of soil water, especially in dry or shallow soils. Thus, we argue that the extent to which DH trees exploit deeper soil water sources is limited, and instead, they on lateral versus vertical soil water exploitation. The DL trees likely do not access deeper soil water nor groundwater, but rather maintained stable and high Ψ_PD_ because of their large water storage capacity. This means that DL trees are not only shallowly rooted, but also dependent on pulse water (Schwinning & Ehleringer, 2010).

In terms of water source shifts, we found evidence that EH species, and select DH species to some extent, exploit other water sources. Into the dry season, EH shifted to lighter δ^18^O and δ^2^H. This change could reflect the utilization of sources such as pockets of water found in relatively shallow rock fractures and in unconsolidated bedrock, as other groundwater sources are likely too deep for roots to access (Frischkorn et al., 1989; Werner & Gerstengarbe, 2003). These localized water sources would move δ^18^O values closer to those of rainwater, that is, not evaporatively enriched. Indeed, two *Z. joazeiro* and one *C. flexuosa* individuals were found relatively close to a small ephemeral drainage area, where water pockets may be found. There is evidence of more deeply rooted evergreen trees from other SDTFs as well. In Mexico, Hasselquist et al. (2010) found that, based on stem water δ^18^O, EH species likely used deeper soil water even though leaf water potentials did not differ from those of deciduous PFTs. Other studies show that evergreen lianas (e.g., *C. flexuosa*) are more aggressive water users (Guzman et al., 2017) and may rely on deeper soil water sources during the dry season (Andrade et al., 2005), although more recent studies refute rooting depth differences in deciduous trees versus lianas (Smith‐Martin et al., 2020). Our own findings are limited, however, because we measured only two EH species, including just one liana individual.

Based on relatively limited vertical root partitioning of soil water, we argue that the difference between the Caatinga PFTs’ abilities to utilize shallow soil water into the dry season depends more on their ability to tolerate high soil water potentials, and their differences in phenological timing. This apparent shallow rooting depth means that the Caatinga represents a model biome to study the role of stem succulence and root function in meeting plant water demands. For example, arbuscular mycorrhizal fungi in the rhizosphere of trees may enhance drought tolerance and could explain in part why DH and EH trees are able to remain active during the dry season while DL are dormant. These are thought to be more infective during the dry season (Teixeira‐Rios et al., 2018) and may not only improve photosynthetic energy use efficiency but also decrease foliar construction cost for some EH species (e.g., *C. flexuosa*), especially after prolonged water deficit (Barros et al., 2018). Future research could adopt the PFT framework to examine the role of root symbionts in drought tolerance, as well as to investigate other plant hydraulic traits across the root, wood, and leaf spectra.

## 5 CONCLUSIONS

Our results broadly support the plant functional type framework as a way to understand water use strategies in SDTFs. Our findings underline the importance of temporal partitioning of water use in the Caatinga, evident in differential drought tolerance levels as a function of phenology and wood density. We found that deciduous low wood density species are generally drought avoiders that employ acquisitive strategies during the wet season. They maintain high leaf water potentials and have relatively low WUE, but their dependence on shallow water sources indicates that stomata are likely sensitive to moderate water stress. Shedding of leaves and of fine roots is initiated before water stress becomes critical, decoupling leaf water potential from drying soil. We found that deciduous high wood density species are moderately drought tolerant. They experience much lower leaf water potentials, have variable WUE, and rely on shallow soil water, including lateral exploration. Deciduous high wood density species are tardily deciduous because their water‐stress tolerance is generally greater than that of deciduous low wood density species. Finally, we found that the evergreen high wood density species are the most drought tolerant. They tolerate very low leaf water potentials, have the highest WUE, and may access alternative water sources. Therefore, we hypothesize that Earth Systems Models which utilize PFTs to assign tree hydraulic traits could perform well in SDTFs (Gillison, 2019; Koven et al., 2020). Still, species‐level violations point to the need for further characterization of tree hydraulic traits and response, such as those related to wood anatomy and root morphology, as well as transpiration, hydraulic conductance, hydraulic safely margins, and hydraulic segmentation, to better constrain our understanding. Characterizing potentially divergent hydraulic strategies within PFTs and among species will be essential for predicting forest responses, mortality, and community composition, especially under extreme drought. SDTFs like the Caatinga could be a model testbed for such studies.

## CONFLICT OF INTEREST

The authors declare no conflict of interest.

## AUTHOR CONTRIBUTIONS

**Cynthia L. Wright:** Conceptualization (equal); writing—original draft (lead); writing—reviewing and editing (lead). **André L. A. de Lima:** Conceptualization (supporting); writing—original draft (supporting); writing‐reviewing and editing (supporting). **Eduardo S. de Souza:** Conceptualization (supporting); writing—original draft (supporting); writing—reviewing and editing (supporting). **Jason B. West:** Conceptualization (equal); writing—original draft (supporting); writing—reviewing and editing (supporting). **Bradford P. Wilcox:** Conceptualization (equal); writing—original draft (supporting); writing—reviewing and editing (supporting).

## Supporting information

Supplementary Material

## Data Availability

Data are available from the Dryad Digital Repository https://doi.org/10.5061/dryad.q2bvq83j6.
