# Supplementary material for: Plant functional types broadly describe water use strategies in the Caatinga, a seasonally dry tropical forest in northeast Brazil
Source: Ecol Evol. 2021 Aug 4;11(17):11808–25. doi: 10.1002/ece3.7949 (PMC8427645; doi:10.1002/ece3.7949)
Supplement: Supplementary file 1 — Supplementary Material [file ECE3-11-11808-s001.docx]

**Supplemental Material**

Table S1. Textural properties of soils at the study site.

| Depth (cm) | Sand  (%) | Clay  (%) | Textural Class | Min. θ (m^3^/m^3^) | Max θ  (m^3^/m^3^) |
| --- | --- | --- | --- | --- | --- |
| 0–10 | 68.5 | 6.4 | sandy loam | 0.039 | 0.548 |
| 10–20 | 64.5 | 11.3 | sandy loam | 0.039 | 0.477 |
| 20–30 | 61.8 | 13.4 | sandy loam | 0.039 | 0.470 |
| 30–40 | 62.7 | 13.3 | sandy loam | 0.039 | 0.438 |
| 40–50 | 62.6 | 15.0 | sandy loam | 0.039 | 0.437 |

**Table S2.** Sample size by date, parameter and species. See Table 2 for species abbreviations.

| **Date** | **4/10/18** | **4/11/18** | **4/28/18** | **5/12/18** | **5/26/18** | **6/9/18** | **6/12/18** | **6/22/18** | **7/7/18** | **7/21/18** | **8/4/18** | **8/22/18** | **9/19/18** | ***Total*** |
| --- | --- | --- | --- | --- | --- | --- | --- | --- | --- | --- | --- | --- | --- | --- |
| Ψ_PD_ | *NC* | *43* | *42* | *42* | *41* | *41* | *NC* | *29* | *33* | *30* | *17* | *18* | *NL* | *336* |
| **AMCE** | NC | 3 | 3 | 3 | 3 | 3 | NC | 2 | 1 | NL | NL | NL | NL | *18* |
| **COLE** | NC | 3 | 3 | 3 | 3 | 3 | NC | 3 | 3 | 3 | 1 | NL | NL | *25* |
| **JAMO** | NC | 3 | 3 | 3 | 3 | 2 | NC | NL | NL | NL | NL | NL | NL | *14* |
| **MAEP** | NC | 3 | 3 | 3 | 2 | 3 | NC | NA | NL | NL | NL | NL | NL | *14* |
| **PSSP** | NC | 3 | 3 | 3 | 3 | 2 | NC | 1 | NL | NL | NL | NL | NL | *15* |
| **SPTU** | NC | 2 | 2 | 2 | 2 | 2 | NC | 2 | 2 | 2 | 1 | 2 | NL | *19* |
| **ANCO** | NC | 3 | 3 | 3 | 3 | 3 | NC | 1 | 3 | 3 | 2 | 2 | NL | *26* |
| **ASPY** | NC | 3 | 3 | 3 | 3 | 3 | NC | 3 | 3 | 3 | 3 | 3 | NL | *30* |
| **BACH** | NC | 3 | 3 | 3 | 3 | 3 | NC | 3 | 3 | 3 | 2 | 1 | NL | *27* |
| **CEPY** | NC | 3 | 2 | 3 | 3 | 3 | NC | 3 | 3 | 2 | NA | 2 | NL | *24* |
| **CRBL** | NC | 3 | 3 | 3 | 3 | 3 | NC | 2 | 3 | NA | NL | NL | NL | *20* |
| **ENSP** | NC | 2 | 2 | 2 | 1 | 1 | NC | 2 | 2 | 2 | 1 | 1 | NL | *16* |
| **MYUR** | NC | 3 | 3 | 3 | 3 | 3 | NC | 3 | 3 | 3 | 2 | 2 | NL | *28* |
| **PIST** | NC | 3 | 3 | 3 | 3 | 3 | NC | 1 | 3 | 3 | 2 | 1 | NL | *25* |
| **CYFL** | NC | 1 | 1 | 1 | 1 | 1 | NC | 1 | 1 | 3 | 1 | 2 | NL | *13* |
| **ZIJO** | NC | 2 | 2 | 1 | 2 | 3 | NC | 2 | 3 | 3 | 2 | 2 | NL | *22* |
| Ψ_MD_ | *NC* | *42* | *42* | *42* | *41* | *39* | *NC* | *27* | *35* | *23* | *20* | *18* | *NL* | *329* |
| **AMCE** | NC | 3 | 3 | 3 | 3 | 3 | NC | 2 | 1 | NL | NL | NL | NL | *18* |
| **COLE** | NC | 3 | 3 | 3 | 3 | 3 | NC | 3 | 3 | 3 | 1 | NL | NL | *25* |
| **JAMO** | NC | 3 | 3 | 3 | 3 | 2 | NC | NL | NL | NL | NL | NL | NL | *14* |
| **MAEP** | NC | 3 | 3 | 3 | 2 | 3 | NC | NA | NL | NL | NL | NL | NL | *14* |
| **PSSP** | NC | 3 | 3 | 3 | 3 | 2 | NC | 1 | NL | NL | NL | NL | NL | *15* |
| **SPTU** | NC | 2 | 2 | 2 | 2 | 2 | NC | 2 | 2 | 2 | 2 | 2 | NL | *20* |
| **ANCO** | NC | 3 | 3 | 3 | 3 | 3 | NC | 1 | 4 | 1 | 2 | 2 | NL | *25* |
| **ASPY** | NC | 3 | 3 | 3 | 3 | 3 | NC | 3 | 3 | 3 | 3 | 3 | NL | *30* |
| **BACH** | NC | 3 | 3 | 3 | 3 | 3 | NC | 3 | 3 | 3 | 2 | 1 | NL | *27* |
| **CEPY** | NC | 3 | 2 | 3 | 3 | 3 | NC | 3 | 3 | 1 | 1 | 2 | NL | *24* |
| **CRBL** | NC | 3 | 3 | 3 | 3 | 3 | NC | 1 | 3 | NA | NL | NL | NL | *19* |
| **ENSP** | NC | 2 | 2 | 2 | 1 | 1 | NC | 2 | 2 | 1 | 2 | 2 | NL | *17* |
| **MYUR** | NC | 3 | 3 | 3 | 3 | 3 | NC | 3 | 3 | 3 | 3 | 2 | NL | *29* |
| **PIST** | NC | 2 | 3 | 3 | 3 | 2 | NC | 1 | 3 | 2 | 1 | 1 | NL | *21* |
| **Table S2, continued.** Sample size by date, parameter and species. See Table 2 for species abbreviations. | | | | | | | | | | | | | | |
| **CYFL** | NC | 1 | 1 | 1 | 1 | 1 | NC | 1 | 2 | 1 | 2 | 1 | NL | *12* |
| **ZIJO** | NC | 2 | 2 | 1 | 2 | 2 | NC | 1 | 3 | 3 | 1 | 2 | NL | *19* |
| δ^13^C | *NC* | *NC* | *43* | *43* | *15* | *42* | *NC* | *38* | *33* | *31* | *26* | *21* | *NL* | *292* |
| **AMCE** | NC | NC | 3 | 3 | NA | 3 | NC | NC | NC | NL | NL | NL | NL | *13* |
| **COLE** | NC | NC | 3 | 3 | 3 | 3 | NC | NC | NC | NC | NC | NL | NL | *22* |
| **JAMO** | NC | NC | 3 | 3 | 2 | 2 | NC | NL | NL | NL | NL | NL | NL | *10* |
| **MAEP** | NC | NC | 3 | 3 | 1 | 3 | NC | NC | NL | NL | NL | NL | NL | *11* |
| **PSSP** | NC | NC | 3 | 3 | 3 | 3 | NC | NC | NL | NL | NL | NL | NL | *14* |
| **SPTU** | NC | NC | 2 | 2 | NA | 2 | NC | NC | NC | NC | NC | NC | NL | *16* |
| **ANCO** | NC | NC | 3 | 3 | 3 | 3 | NC | NC | NC | NC | NC | NC | NL | *27* |
| **ASPY** | NC | NC | 3 | 3 | 3 | 3 | NC | NC | NC | NC | NC | NC | NL | *27* |
| **BACH** | NC | NC | 3 | 3 | NA | 3 | NC | NC | NC | NC | NC | NC | NL | *21* |
| **CEPY** | NC | NC | 3 | 3 | NA | 3 | NC | NC | NC | NC | NC | NC | NL | *23* |
| **CRBL** | NC | NC | 3 | 3 | NA | 3 | NC | NC | NC | NC | NL | NL | NL | *16* |
| **ENSP** | NC | NC | 2 | 2 | NA | 1 | NC | NC | NC | NC | NC | NC | NL | *15* |
| **MYUR** | NC | NC | 3 | 3 | NA | 3 | NC | NC | NC | NC | NC | NC | NL | *23* |
| **PIST** | NC | NC | 3 | 3 | NA | 3 | NC | NC | NC | NC | NC | NC | NL | *21* |
| **CYFL** | NC | NC | 1 | 1 | NA | 1 | NC | NC | NC | NC | NC | NC | NL | *11* |
| **ZIJO** | NC | NC | 2 | 2 | NA | 3 | NC | NC | NC | NC | NC | NC | NL | *22* |
| δ^2^H | *42* | *NC* | *NC* | *NC* | *NC* | *NC* | *44* | *NC* | *NC* | *NC* | *NC* | *NC* | *5* | *91* |
| **AMCE** | 3 | NC | NC | NC | NC | NC | NC | NC | NC | NL | NL | NL | NL | *6* |
| **COLE** | 3 | NC | NC | NC | NC | NC | NC | NC | NC | NC | NC | NL | NL | *6* |
| **JAMO** | 3 | NC | NC | NC | NC | NC | NC | NL | NL | NL | NL | NL | NL | *6* |
| **MAEP** | 3 | NC | NC | NC | NC | NC | NC | NC | NL | NL | NL | NL | NL | *6* |
| **PSSP** | 3 | NC | NC | NC | NC | NC | NC | NC | NL | NL | NL | NL | NL | *6* |
| **SPTU** | 2 | NC | NC | NC | NC | NC | NC | NC | NC | NC | NC | NC | NL | *4* |
| **ANCO** | 3 | NC | NC | NC | NC | NC | NC | NC | NC | NC | NC | NC | NL | *6* |
| **ASPY** | 3 | NC | NC | NC | NC | NC | NC | NC | NC | NC | NC | NC | NL | *6* |
| **BACH** | 3 | NC | NC | NC | NC | NC | NC | NC | NC | NC | NC | NC | NL | *6* |
| **CEPY** | 3 | NC | NC | NC | NC | NC | NC | NC | NC | NC | NC | NC | NL | *6* |
| **CRBL** | 2 | NC | NC | NC | NC | NC | NC | NC | NC | NC | NL | NL | NL | *5* |
| **ENSP** | 2 | NC | NC | NC | NC | NC | NC | NC | NC | NC | NC | NC | NL | *4* |
| **MYUR** | 3 | NC | NC | NC | NC | NC | NC | NC | NC | NC | NC | NC | NL | *6* |
| **PIST** | 3 | NC | NC | NC | NC | NC | NC | NC | NC | NC | NC | NC | NL | *6* |
| **CYFL** | 1 | NC | NC | NC | NC | NC | NC | NC | NC | NC | NC | NC | NL | *4* |
| **ZIJO** | 2 | NC | NC | NC | NC | NC | NC | NC | NC | NC | NC | NC | NL | *8* |
| **Table S2, continued.** Sample size by date, parameter and species. See Table 2 for species abbreviations. | | | | | | | | | | | | | | |
| δ^18^O | *42* | *NC* | *NC* | *NC* | *NC* | *NC* | *44* | *NC* | *NC* | *NC* | *NC* | *NC* | *5* | *91* |
| **AMCE** | 3 | NC | NC | NC | NC | NC | NC | NC | NC | NL | NL | NL | NL | *6* |
| **COLE** | 3 | NC | NC | NC | NC | NC | NC | NC | NC | NC | NC | NL | NL | *6* |
| **JAMO** | 3 | NC | NC | NC | NC | NC | NC | NL | NL | NL | NL | NL | NL | *6* |
| **MAEP** | 3 | NC | NC | NC | NC | NC | NC | NC | NL | NL | NL | NL | NL | *6* |
| **PSSP** | 3 | NC | NC | NC | NC | NC | NC | NC | NL | NL | NL | NL | NL | *6* |
| **SPTU** | 2 | NC | NC | NC | NC | NC | NC | NC | NC | NC | NC | NC | NL | *4* |
| **ANCO** | 3 | NC | NC | NC | NC | NC | NC | NC | NC | NC | NC | NC | NL | *6* |
| **ASPY** | 3 | NC | NC | NC | NC | NC | NC | NC | NC | NC | NC | NC | NL | *6* |
| **BACH** | 3 | NC | NC | NC | NC | NC | NC | NC | NC | NC | NC | NC | NL | *6* |
| **CEPY** | 3 | NC | NC | NC | NC | NC | NC | NC | NC | NC | NC | NC | NL | *6* |
| **CRBL** | 2 | NC | NC | NC | NC | NC | NC | NC | NC | NC | NL | NL | NL | *5* |
| **ENSP** | 2 | NC | NC | NC | NC | NC | NC | NC | NC | NC | NC | NC | NL | *4* |
| **MYUR** | 3 | NC | NC | NC | NC | NC | NC | NC | NC | NC | NC | NC | NL | *6* |
| **PIST** | 3 | NC | NC | NC | NC | NC | NC | NC | NC | NC | NC | NC | NL | *6* |
| **CYFL** | 1 | NC | NC | NC | NC | NC | NC | NC | NC | NC | NC | NC | NL | *4* |
| **ZIJO** | 2 | NC | NC | NC | NC | NC | NC | NC | NC | NC | NC | NC | NL | *8* |

NC means no collection was made, although a collection could have been possible.

NL means there was no leaf cover, so a collection was not possible.

NA means the sample was not analyzed because it was damaged.

| 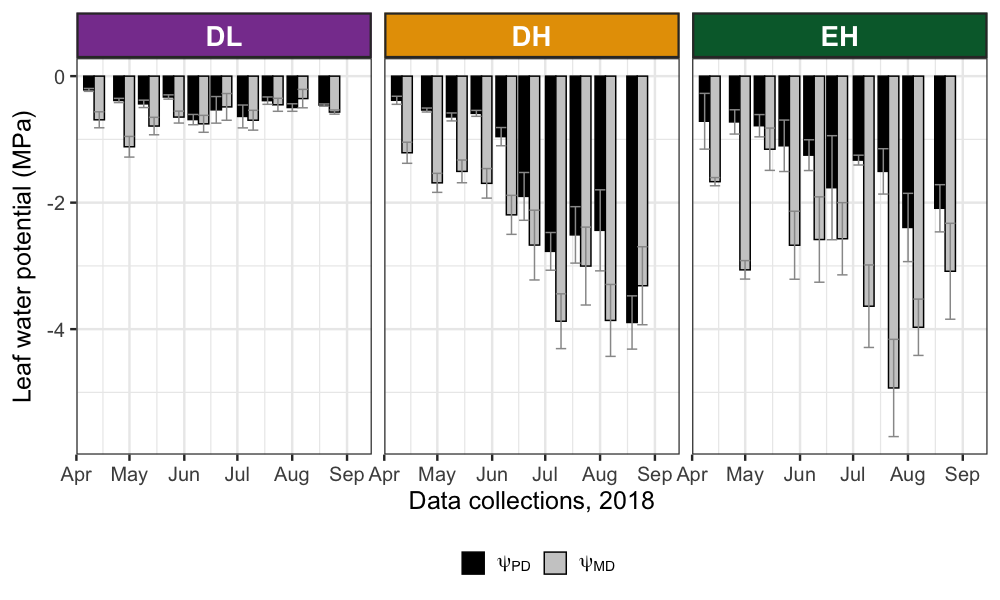 |
| --- |
| **Figure S1.** Leaf water potentials for the three plant functional types measured biweekly from April 11to August 22, 2018. Error bars are standard deviations. See Table 2 for PFT color-code. |

| 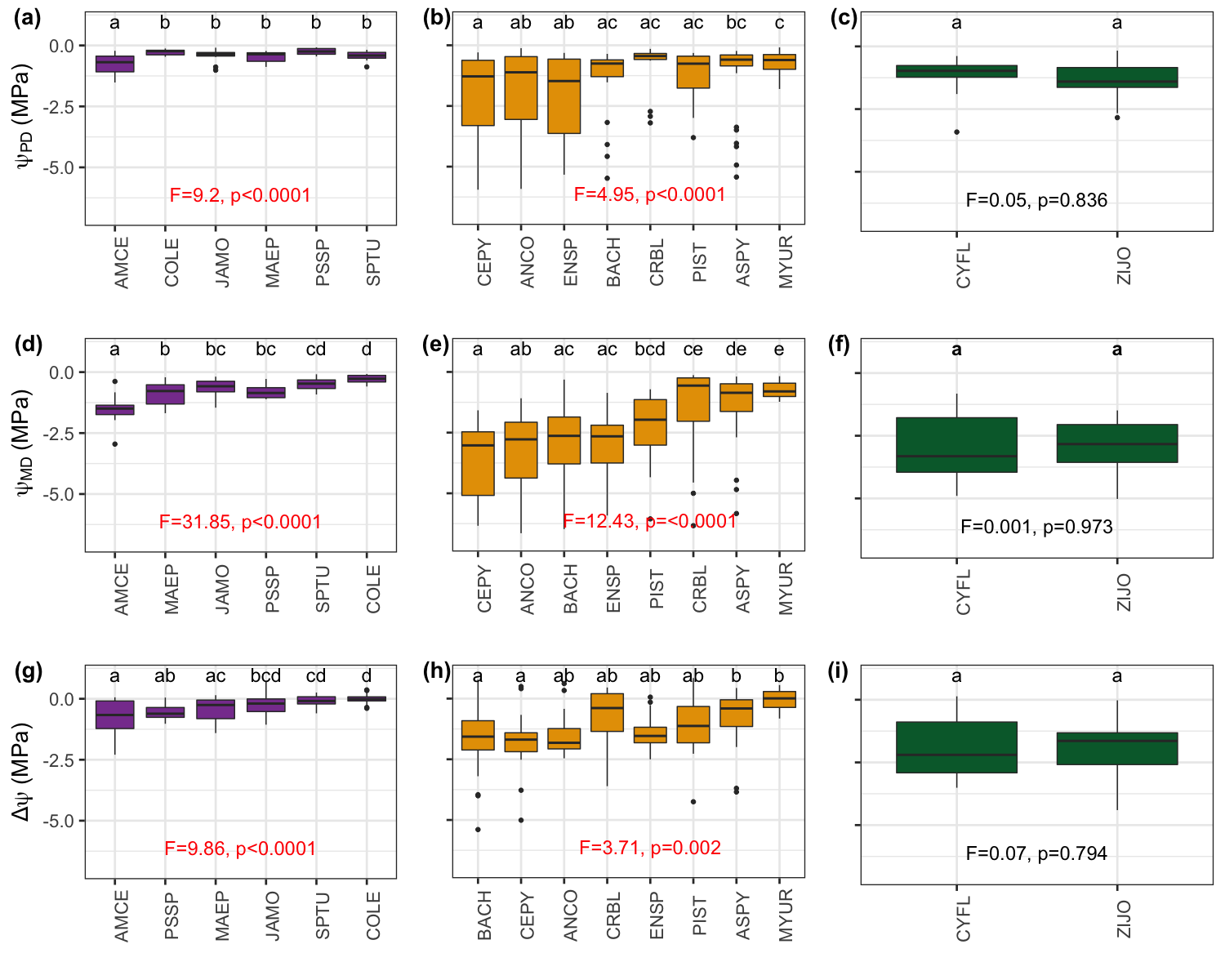 |
| --- |
| **Figure S2.** Averaged leaf water potentials by species for the period April 11–August 22, 2018: pre-dawn (Ψ_PD_), mid-day (Ψ_MD_), and the difference between them (ΔΨ). Error bars are standard deviations. Significant linear mixed effects model results are denoted by the F statistics and p-values shown in red. Letters denote pair-wise comparisons from Tukey’s post-hoc procedure. See Table 2 for PFT color-code. |

| *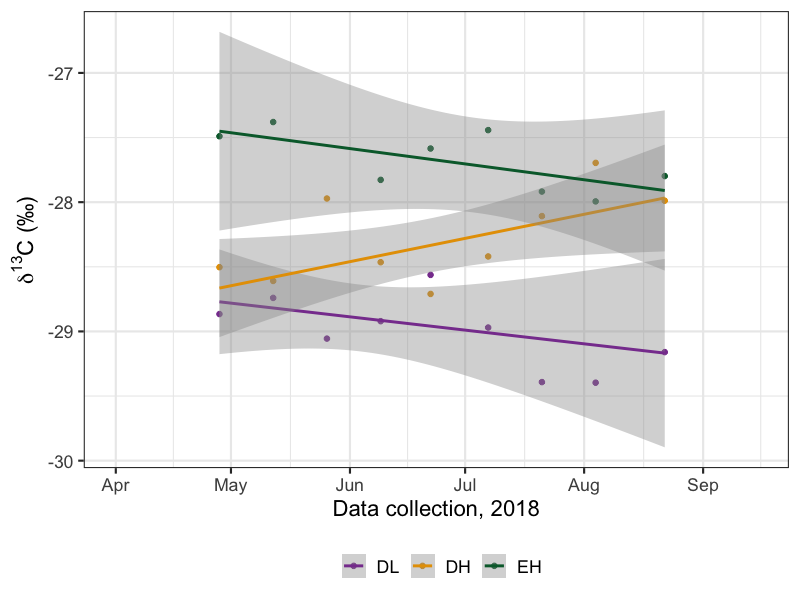* |
| --- |
| Figure S3. Trends in foliar δ^13^C, measured from April 28 to August 22, 2018. Grey shading depicts the standard deviation associated with the linear regression fit. See Table 2 for PFT color-code. |

| 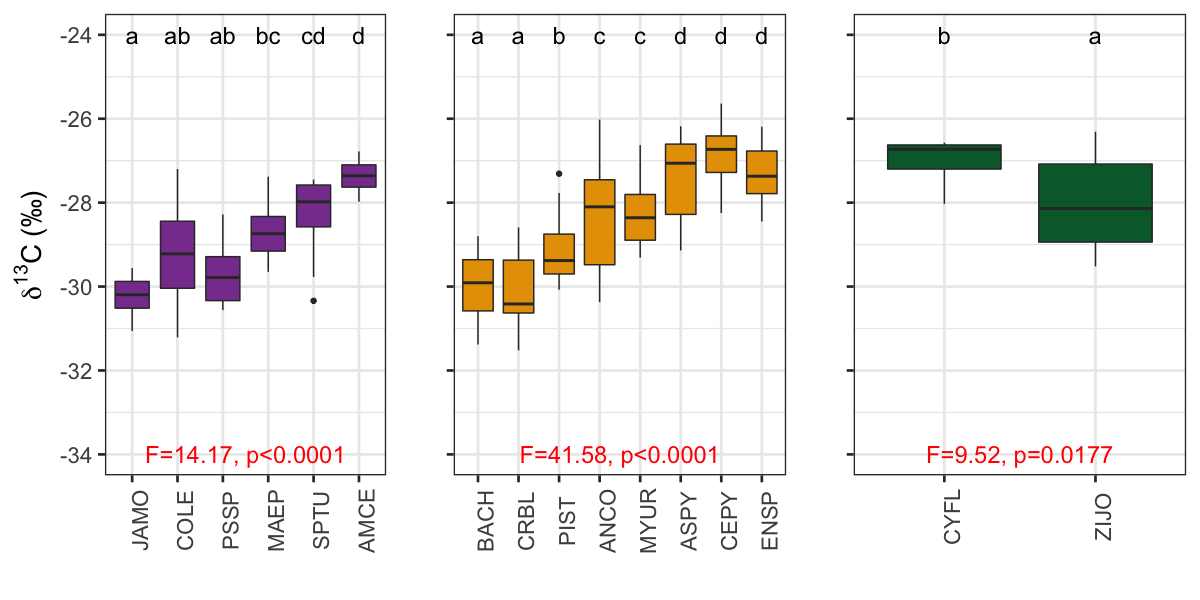 |
| --- |
| Figure S4. Species-level variations in average foliar δ^13^C within each for the period April 28 to August 22, 2018. Error bars are standard deviations. Significant linear mixed effects model results are denoted by the F statistics and p-values shown in red. Letters denote pair-wise comparisons from Tukey’s post-hoc procedure. See Table 2 for PFT color-code and for species abbreviations. |

| (a)  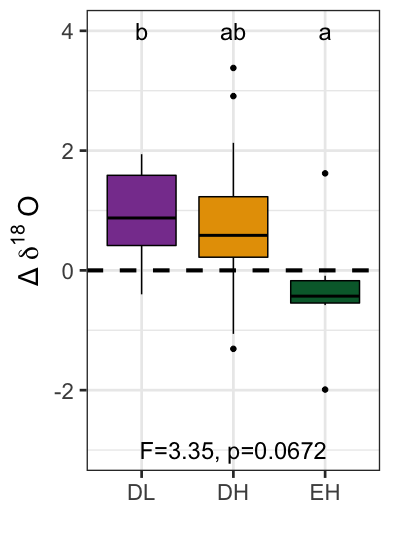 | (b)  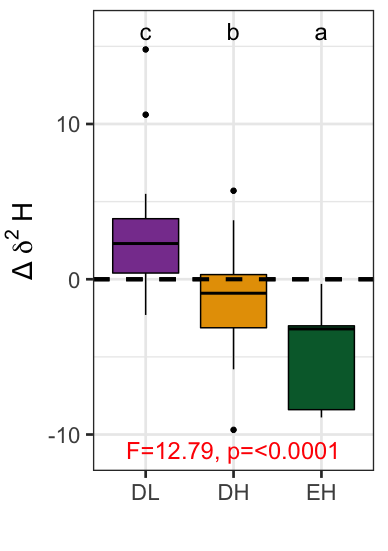 |
| --- | --- |
| **Figure S5. S**hift in stem water isotopic composition as (a) Δδ^18^O and (b) Δδ^2^H between June and April collections, summarized by PFT. Error bars are standard deviations, and the dashed line indicates no change. Linear mixed effect model results for PFTs are denoted by the F statistics and p-values, with significant differences in red. Letters denote statistical groupings based Tukey’s post-hoc. See Table 2 for PFT color-code and species abbreviations. | |

| 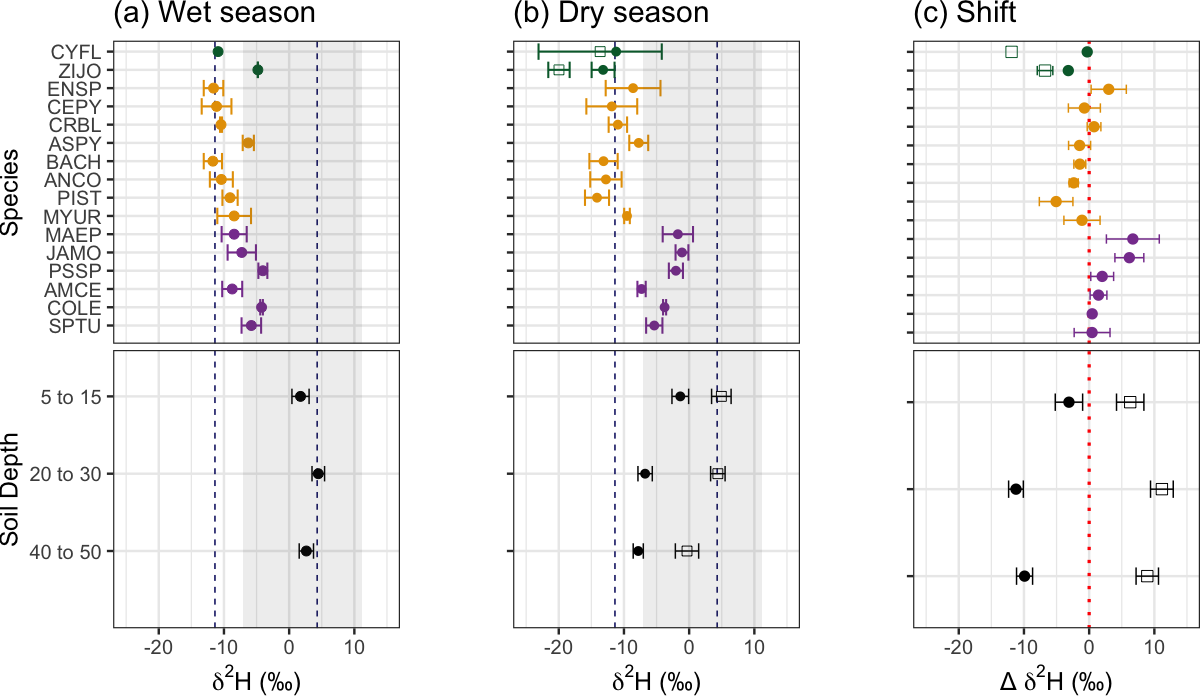 |
| --- |
| **Figure S6.** Stem and soil water δ^2^H for (a) the wet season (solid point: April 10, 2018); (b) the dry season (solid point: June 12, 2018; empty square: Sept. 19, 2018 for EH only); and (c) the shift between sampling dates, Δ δ^18^O (solid point: difference between June and April; empty square: difference between Sept. and June for EH only). For reference, the average ± standard deviation of the rainwater (grey shading) and groundwater (dashed lines) are also shown. See Table 2 for PFT color-code and species abbreviations. |
